# Identification of Regulatory Networks of MicroRNAs and Their Targets in Response to *Colletotrichum gloeosporioides* in Tea Plant (*Camellia sinensis* L.)

**DOI:** 10.3389/fpls.2019.01096

**Published:** 2019-09-12

**Authors:** Anburaj Jeyaraj, Xuewen Wang, Shuangshuang Wang, Shengrui Liu, Ran Zhang, Ailin Wu, Chaoling Wei

**Affiliations:** ^1^State Key Laboratory of Tea Plant Biology and Utilization, Anhui Agricultural University, Hefei, China; ^2^Department of Biotechnology, Karpagam Academy of Higher Education, Coimbatore, India; ^3^Department of Genetics, University of Georgia, Athens, United States

**Keywords:** *Colletotrichum gloeosporioides*, *Camellia sinensis*, microRNA, regulatory network, degradome

## Abstract

Anthracnose disease is caused by *Colletotrichum gloeosporioides*, and is common in leaves of the tea plant (*Camellia sinensis*). MicroRNAs (miRNAs) have been known as key modulators of gene expression in response to environmental stresses, disease resistance, defense responses, and plant immunity. However, the role of miRNAs in responses to *C. gloeosporioides* remains unexplored in tea plant. Therefore, in the present study, six miRNA sequencing data sets and two degradome data sets were generated from *C. gloeosporioides*-inoculated and control tea leaves. A total of 485 conserved and 761 novel miRNAs were identified. Of those, 239 known and 369 novel miRNAs exhibited significantly differential expression under *C. gloeosporioides* stress. One thousand one hundred thirty-four and 596 mRNAs were identified as targets of 389 conserved and 299 novel miRNAs by degradome analysis, respectively. Based on degradome analysis, most of the predicted targets are negatively correlated with their corresponding conserved and novel miRNAs. The expression levels of 12 miRNAs and their targets were validated by quantitative real-time PCR. A negative correlation between expression profiles of five miRNAs (PC-5p-80764_22, csn-miR160c, csn-miR828a, csn-miR164a, and csn-miR169e) and their targets (WRKY, ARF, MYB75, NAC, and NFY transcription factor) was observed. The predicted targets of five interesting miRNAs were further validated through 5’RLM-RACE. Furthermore, Gene Ontology and metabolism pathway analysis revealed that most of the target genes were involved in the regulation of auxin pathway, ROS scavenging pathway, salicylic acid mediated pathway, receptor kinases, and transcription factors for plant growth and development as well as stress responses in tea plant against *C. gloeosporioides* stress. This study enriches the resources of stress-responsive miRNAs and their targets in *C. sinensis* and thus provides novel insights into the miRNA-mediated regulatory mechanisms, which could contribute to the enhanced susceptibility of *C. gloeosporioides* in tea plant.

## Introduction

Plants have evolved to respond to biotic and abiotic stresses through a repertoire of mechanisms, which regulate gene expression to maximize chances of survival in hostile conditions (Dorantes-Acosta et al., 2012). Under biotic stresses, plants trigger two layers of immunity against pathogens, which are pathogen-associated molecular patterns (PAMPs) triggered immunity (PTI) and effector-triggered immunity (ETI). Pathogenic organisms are recognized by conserved PAMPs (elicitors) through pattern-recognition receptors (PRRs) located on the cell surface. Perception of PAMPs triggers basal defense, also known as PTI, which encompasses the immune responses against most pathogens (Jones and Dangl, 2006). Effective pathogens have evolved mechanisms to counteract the basal defense by delivering PTI interfering effector proteins into the plant cells (Chisholm et al., 2006). In turn, many plants have evolved another layer of immunity, ETI. The ETI response is facilitated by proteins encoded by resistance (R) genes, which are typically nucleotide-binding site leucine-rich repeat (NBS-LRR) proteins (Cui et al., 2015). Most studies of plant immunity have focused on the transcriptional regulation of protein-coding genes. Recently, it has been found that diverse miRNAs are responsive to infection and stress, and function in plant responses to both biotic and abiotic stresses (Xin et al., 2010; Chen et al., 2017).

In recent years, several studies have shown that small non-protein-coding RNAs (sRNAs) are key molecules that regulate diverse eukaryotic biological processes, including transcription, oxidation-reduction, transport, and stress response. Plant microRNAs (miRNAs) and small interfering RNAs (siRNAs) are two major classes of sRNAs and are classified according to their biogenesis (Jones-Rhoades et al., 2006). MiRNAs are derived from single-stranded stem-loop precursor structures, and siRNAs are processed from perfect double-stranded RNA transcripts (Peters and Meister, 2007). Plant miRNAs are an extensive class of newly discovered endogenous small regulatory RNA molecules, which are 20–24 nucleotides in length and negatively regulate gene expression at the post-transcriptional level by guiding target mRNA cleavage or translation inhibition (Jones-Rhoades et al., 2006). MiRNAs play an important role in responses to biotic and abiotic stressors through their interactions with their target mRNAs (Chen et al., 2017). For example, miR393 represses auxin signaling to promote bacterial PAMP-triggered immunity (PTI) (Navarro et al., 2006).

The rapid advances in high-throughput sequencing technologies as well as bioinformatics analyses have provided a highly efficient strategy for discovering conserved and novel miRNAs in several plant species (Xu et al., 2012, Chen et al., 2017), despite the unavailability of plant whole genomic sequences (Xu et al., 2012). To date, 38,589 precursor miRNAs and 48,885 mature miRNAs from 271 species have been deposited in the miRNA database miRBase (Release 22, www.mirbase.org) (Kozomara and Griffiths-Jones, 2014). However, several studies of miRNAs were previously reported (Zhang et al., 2014; Zheng et al., 2015; Liu et al., 2016; Jeyaraj et al., 2017). Tea genome sequence information was recently released by Wei et al. (2018). Identification of miRNA target genes is critical for the elucidation of the pathways they regulate. Degradome sequencing of mRNA combined with gene functional annotation can predict and verify target genes of miRNAs in many plants (Addo-Quaye et al., 2008). This approach for identifying miRNAs and their targets firmly depends on the availability of genomic information and miRNA databases (e.g., miRBase).

The tea plant [*Camellia sinensis* (L.) O. Kuntze] is an economically important and evergreen woody perennial plantation crop that grows mainly in tropical and subtropical climates (Mukhopadhyay et al., 2016). Tea is one of the most popular non-alcoholic beverages in the world and is consumed by over two-thirds of the world population. The tea plant is susceptible to biotic (bacterial, fungal, and viral diseases) and abiotic (cold and drought) stresses (Deng et al., 2013). *Colletotrichum gloeosporioides* is considered to be one of the dominant endophytic taxa of *C. sinensis*, which leads to the fungal disease anthracnose (Fang et al., 2013). As the disease progresses, yellow oval spots will turn to concentric brown rings with scattered dots; it eventually leads to defoliation, affecting both young and old leaves. Unfortunately, many tea plant cultivars are highly susceptible to anthracnose disease, which causes severe damage accompanied by high yield losses (Fang et al., 2013). It was recently reported that symptoms of blight on the leaves of anthracnose infected plants were detected in 30–60% of the *C. sinensis* fields in the Yellow Mountain region in Anhui province of China (Guo et al., 2014).

The infection of hemibiotrophic fungus (*C. gloeosporioides*) displays an initial biotrophic phase, followed by a necrotrophic stage (Münch et al., 2008). The salicylic acid (SA), jasmonic acid (JA), and ethylene (ET) signaling pathway is activated against necrotrophic as well as hemibiotrophic pathogens. The SA analog BTH (benzo thiadiazole-7-carbothioic acid S-methyl ester) treatment triggered the accumulation of SA-inducible defense proteins, which in turn activate defense-related genes during anthracnose infection in cowpea seedlings (Latunde-Dada and Lucas, 2001) and cucumber (Deepak et al., 2006). However, the miRNA-mediated gene regulation in response to *C. gloeosporioides* is unknown.

In tea plant, a fewer number of conserved and novel miRNAs were identified relative to other model plants (Zhu and Luo, 2013). Recently, a limited number of tea miRNAs were identified from responses to environmental stresses, such as drought, cold, and insect-induced stress (Zhang et al., 2014; Zheng et al., 2015; Liu et al., 2016; Jeyaraj et al., 2017). However, the miRNA-mediated gene regulatory networks that respond to the biotic *C. gloeosporioides* stress remain unexplored in tea plant. Therefore, in the present study, we employed high-throughput sRNA sequencing technology to identify miRNAs from healthy leaves (the control CK) and leaves treated with *C. gloeosporioides*. Differentially expressed miRNAs between CK and the biotic treatment were identified, and further potential targets of miRNAs were predicted through degradome sequencing. Biological functions of target genes were analyzed along with their interactions with their respective miRNAs, and results were validated by quantitative real-time polymerase chain reaction (qRT-PCR). The predicted cleavage sites in target gene mRNAs of five csn-miRNAs were validated through 5’RLM-RACE. These results lay the foundation for understanding the regulation of miRNAs and their respective target genes in response to *C. gloeosporioides* stress in tea plant.

## Materials and Methods

### Plant Growth Conditions

Tea plant (*C. sinensis* L. var. Shuchazao) used in the present study were grown in the tea plantation at Anhui Agricultural University, Hefei, China. Three-year-old cuttings were planted in pots (30-cm diameter, 35-cm height) and grown in a green house maintained at 23 ± 3°C with 65 ± 5% room humidity and a 16/8 h (day/night) photoperiod. All experimental plants were irrigated once a day and fertilized once a month. Healthy Shuchazao cuttings with uniform growth (25–30 cm in height) were selected for experiments.

### Isolation of Fungal Pathogen

Diseased leaves of Shuchazao were collected from the tea plantation, which is located at Anhui Agricultural University, Hefei City, Anhui Province in China. The pathogenic *C. gloeosporioides* was originally isolated from diseased leaves showing visible anthracnose symptoms. Briefly, the infected leaves with the area brown lesions were cut into small pieces. These small pieces were surface sterilized in 2% NaClO for 3 min, followed by 70% ethanol for 1 min, rinsed twice in sterile water, and then transferred to potato dextrose agar (PDA) containing plate. The culture was incubated at 28°C for 5 days. Single germinating spores were picked up with a sterilized needle and transferred to a new PDA plate, and incubation at 28°C was continued to generate the pure isolates (Cai et al., 2009).

### DNA Extraction, PCR Amplification, and Sequencing

The fungal genomic DNA was extracted from fresh mycelia grown in potato dextrose broth (PDB, liquid media) for 5 days, using a modified CTAB protocol as described by Guo et al. (2000). DNA quality and quantity were determined by spectrophotometer and electrophoresis. DNA samples were stored at −80°C for further analysis. In order to identify *C. gloeosporioides*, the ribosomal internal transcribed spacer (ITS) was amplified in a thermocycler (Bio-Rad, Hercules, USA) using ITS1 (5′-TCC GTA GGT GAA CCT GCG G-3′) and ITS4 (5′-TCC TCC GCT TAT TGA TAT GC-3′). The 25 μl of reaction mixture consisted of 12.5 μl of Premix Taq™ (Takara, Dalian, China), 1 μl of forward primer, 1 μl of reverse primer, 2 μl of template DNA, and 8.5 μl of double-distilled water. Amplification of ITS regions was performed using the following PCR reaction conditions of 95°C for 5 min, followed by 35 cycles of 94°C for 45 s, 60°C for 30 s, 72°C for 45 s, and final extension at 72°C for 5 min. The final PCR products were analyzed on 1% agarose gel and purified using AxyPrep DNA gel extraction kit (AxyPrep, Hangzhou, China) according to the manufacturer’s protocol. The purified products were sequenced and analyzed by GenScript (Nanjing, China). To identify the fungus, the resulted sequences were subjected to BLAST search (https://blast.ncbi.nlm.nih.gov/Blast.cgi) with the National Center for Biotechnology Information (NCBI) database. The sequences of ITS region of all isolates had 100% homology with *C. gloeosporioides* (GenBank: JX010223.1) isolates available in the NCBI (Weir et al., 2012).

### Pathogen Inoculations and Treatments

For pathogenicity tests, *C. gloeosporioides* was cultured on PDA and incubated at 25°C ± 2°C in darkness for 10 days to promote sporulation. Conidia were harvested and suspended in sterile distilled water. The conidia concentration was determined using a hemocytometer and adjusted to 1×10^6^ ml^-1^ for inoculation. The third healthy mature leaves from 3-year-old tea plants were surface-sterilized with 75% ethanol and sterile distilled water. The leaves were wounded on the upper surface with a sterile needle, and 20 μl of conidial suspensions was applied to the wound. An equal volume of sterile water was applied as a mock inoculation. After inoculation, each plant was enclosed in a plastic bag to maintain high relative humidity for conidial germination. The *C. gloeosporioides*–inoculated (CgIL) and mock-inoculated control (CK) leaves were harvested at 1, 4, 7, 10 and 13 days after inoculation. All samples were immediately frozen in liquid nitrogen and then stored at −80°C for further use. Three biological replicates were used for each experiment in this study. The samples were assigned a name with the information of treatment type and treated days, i.e., CgIL4d references the samples collected after 4 days of *C. gloeosporioides* treatment. Three experimental replicates were conducted for the treatment and control.

### sRNA Library Construction and High-Throughput Sequencing

Total RNA was isolated from each frozen sample using Trizol reagent (Invitrogen, CA, USA) according to the manufacturer’s protocol. The quantity and quality of the total RNA were determined using a Bioanalyzer 2100 (Agilent, CA, USA) and the RNA 6000 Nano LabChip Kit (Agilent, CA, USA) with RIN number >7.0. To identify early response of tea plant to *C. gloeosporioides* infection, equal quantities of total RNA from *C. gloeosporioides*- and mock-inoculated leaves collected at 4 days post infection (4dpi) were used to prepare the sRNA libraries using the TruSeq Small RNA Sample Prep Kit (Illumina, San Diego, CA, USA). We performed single-end sequencing (36 bp) on an Illumina Hiseq2500 at the LC-BIO (Hangzhou, China) using the vendor’s recommended protocol. The sRNA read data from this study have been deposited in the Gene Expression Omnibus (GEO) database; the accession number is GSE119728 (https://www.ncbi.nlm.nih.gov/geo/query/acc.cgi?acc=GSE119728).

### sRNA Data Analysis and Identification of Conserved and Novel miRNAs

The raw reads generated from the Illumina GAIIX system were processed according to the procedures as described in a previous study (Li et al., 2010) by LC Sciences. In brief, the raw reads were filtered using the Illumina pipeline filter (Solexa 0.3), and then further processed with the in-house program (ACGT101-v4.2-miR, LC Sciences, Houston, Texas, USA) to remove adapter dimers, junk, low complexity reads, common RNA families (rRNA, tRNA, snRNA, snoRNA) and repeats. Subsequently, unique sequences ranging from 18 to 26 nt in length were compared with known plant miRNAs in miRBase (Release 22; http://www.mirbase.org) (Kozomara and Griffiths-Jones, 2014), using BLAST searches to identify known miRNAs with no more than two mismatches. After the analyses, known miRNAs were categorized into four groups (1a, 1b, 2a, and 3a).

The remaining unmatched reads were aligned to a *C. sinensis* genome assembly (Wei et al., 2018) using BLASTn, with no mismatches permitted. The sequences containing hairpin RNA structures were predicated from the flanking 80 nt sequences using RNAfold software (http://unafold.rna.albany.edu/?q=mfold/RNA-Folding-Form) (Zuker, 2003). The following criteria were used for predicting the pre-miRNA secondary structure: i) the number of nucleotides in one bulge in stem was ≤12; ii) the number of base pairs in the stem region of a predicted hairpins was ≥16; iii) the Gibbs free energy (kCal/mol) threshold was ≤−15; iv) the length of the hairpin (up and down stems + terminal loop) was ≥50; v) the length of the hairpin loop was ≤20; vi) the number of nucleotides in one bulge in the mature region was ≤8; vii) the number of biased errors in one bulge in a mature region was ≤4; viii) the number of biased bulges in a mature region was ≤2; ix) the number of errors in a mature region was ≤7; x) the number of base pairs in a mature region of the predicted hairpin was ≥12; and xi) maturity percentage in the stem was ≥80.

The abundances of miRNAs were normalized to reads per million (RPM) for each library. The original counts of miRNA reads were used for differentially expressed miRNAs (DEMs) analysis as reported earlier (Liu et al., 2016). DESequence analysis was conducted to identify the DEMs with changes of at least twofold in abundance (*p* ≤ 0.05), relative to the CK control.

### Degradome Sequencing and Data Analysis

Total RNA was extracted from the samples using Trizol reagent (Invitrogen, CA, USA) according to the manufacturer’s protocols. Total RNA from the three biological replicates of each inoculation time (CK and CgIL) was pooled in equal amounts to generate each degradome library. Two libraries were constructed according to the methods described by Addo-Quaye et al. (2009) and Ma et al. (2010) with minor modifications. Briefly, polyA-enriched RNA was mixed with biotinylated random primers, and then the RNA containing the biotinylated random primers was captured by beads and ligated to 5′ adaptors. The ligated products were used to generate first strand cDNA by reverse transcription. After a short PCR amplification, additional DNA products were produced. Following purification, digestion, ligation, and purification, the cDNA library was sequenced with an Illumina Hiseq2500 (LC-BIO, Hangzhou, China).

Raw sequencing reads were obtained using Illumina’s Pipeline v1.5 software and processed to remove adaptor sequences and low-quality sequencing reads. The unique sequencing reads with lengths of 20 and 21 nt were then used to identify potentially cleaved targets with a public software package (CleaveLand3.0 pipeline), as previously described (Addo-Quaye et al., 2008). The degradome reads were mapped to *C. sinensis* reference sequences, including tea genome scaffolds and contigs from whole-genome shotgun sequencing and assembly, tea transcriptome sequences from the NCBI Sequence Read Archive (SRA, GenBank accession no. SRR1979118), EST sequences, genomic survey sequences (GSSs), and nucleotide sequences downloaded from the GenBank nucleotide databases at NCBI (http://www.ncbi.nlm.nih.gov). The potential target genes of differentially expressed miRNAs were predicted by Target finder. Alignment score was introduced based on the alignment between each miRNA and its potential target. Mismatched pairs or single nucleotide bulges were each scored as 1 and G:U pairs were scored as 0.5. Mismatched and G:U pair scores were doubled within the core segment (nucleotide pairs at positions 2–13). Furthermore, based on the abundances of the degradome sequences and cleavage sequences, the miRNA targets were classified into five categories (0, 1, 2, 3, and 4) according to the CleaveLand 3.0 pipeline with default parameters (Addo-Quaye et al., 2008).

### Functional Analysis of Target Genes and Network Analysis

To better understand the function of the target genes and their corresponding metabolic network during *C. gloeosporioides* stress in tea plant, annotations of target gene functions were performed using the Gene Ontology (GO, http://www.geneontology.org/) and Kyoto Encyclopedia of Genes and Genomes (KEGG) (http://www.genome.jp/kegg/) pathway databases. The most abundant DEM targets were annotated based on sequence similarity by performing a BLAST_X_ search against the GO protein database. Furthermore, target genes were categorized according to their functions under biological processes, molecular functions, and cellular components using GO analysis. The enriched metabolic pathways or signal transduction pathways of potential miRNA target genes were validated using KEGG enrichment analysis to enrich the KEGG terms according to Zhang et al. (2016). Networks between miRNAs and their target genes were subsequently assembled according to the GO analysis results.

### Validation of miRNA Expression Profiles and Their Targets

The expression levels of randomly selected miRNAs and their targets were validated by qRT-PCR. Total RNA was isolated from the samples taken at 1dpi, 4dpi, 7dpi, 10dpi, and 13dpi using Trizol reagent (Invitrogen, CA, USA), and the quantity and purity of the total RNA were determined using a Bioanalyzer 2100 (Agilent, CA, USA) and the RNA 6000 Nano LabChip Kit (Agilent, CA, USA). Five hundred nanograms of total RNA from samples was reverse transcribed to cDNA using PrimeScript™ RT Master Mix (Takara, Dalian, China) according to the manufacturer’s instructions. The first-strand cDNA was used as a template for qRT-PCR with miRNA and target gene specific primers. For qRT-PCR of miRNAs, the stem-loop RT primers, forward primers, and reverse primers were designed for each individual miRNA according to the criteria described by Varkonyi-Gasic et al. (2007). The primers for mRNA qRT-PCR were designed using primer premier 5.0 (http://www.premierbiosoft.com/primerdesign/index.html). The amplification was conducted on the CFX96 real-time detection system (Bio-Rad, Hercules, USA) using SYBR Premix Ex Taq^™^ (Takara, Dalian, China) following the manufacturer’s instructions. U6 small nuclear RNA (U6 snRNA) and glyceraldehyde-3-phosphate dehydrogenase (GADPH) genes were used as the references for qRT-PCR of miRNAs and mRNAs, respectively. The expression levels of miRNAs and target genes were determined by calculating fold change using the 2^–ΔΔCt^ method. All qRT-PCR analyses were performed in three biological replicates, each of which consisted of three technical replicates. Detailed information about the primers used in this study is presented in **Supplementary Table 1**.

### Verification of miRNA Target Genes by 5’RLM-RACE

The cleavage sites of selected miRNA targets were validated through 5’RLM-RACE using the FirstChoice RLM-RACE Kit (Invitrogen, Thermo Fisher Scientific) according to the manufacturer’s protocol. Briefly, 10 μg of total RNA was ligated to the 5’RNA adapter using T4 RNA ligase and reverse transcribed to cDNA. Amplification of cleaved miRNA target gene products was performed using target gene specific reverse primers and RNA adapter specific forward primers (**Supplementary Table 1**). The final RLM-RACE products were analyzed on agarose gels and then purified using the DNA gel extraction kit (Corning Life Sciences, Suzhou, China) according to the manufacturer’s instruction. Purified products were directly cloned into pEASY-T1 vectors (TransGen Biotech, Beijing, China), transformed into *Escherichia coli* Trans1-T1 competent cells (TransGen Biotech) and sequenced. The sequencing results were analyzed to map the cleavage sites. The primers used to amplify cleavage products of tea miRNA target genes through 5’RLM-RACE are listed in **Supplementary Table 1**.

### Statistical Analysis

Fisher’s exact test, chi-squared 2×2 test, chi-squared nXn test, Student’s t test, or ANOVA was performed based on the experimental designs to compare the expression of miRNAs and degradation of target genes between the CK and CgIL libraries based on normalized deep-sequencing counts. The significance threshold was set to be 0.01 and 0.05 in each test, and the log2 ratio was regarded as a threshold to detect fold changes of miRNA expression and target gene degradation. qRT-PCR data obtained in this study are presented as their means ± standard deviation values. The expression profiles of the miRNAs and target genes identified by qRT-PCR were subjected to Duncan’s multiple range tests (DMRT) using DPS software (www.chinadps.net) (Deng et al., 2013).

## Results

### Pathogenic Fungal Identification and Pathogenicity Tests

**Figure 1:**
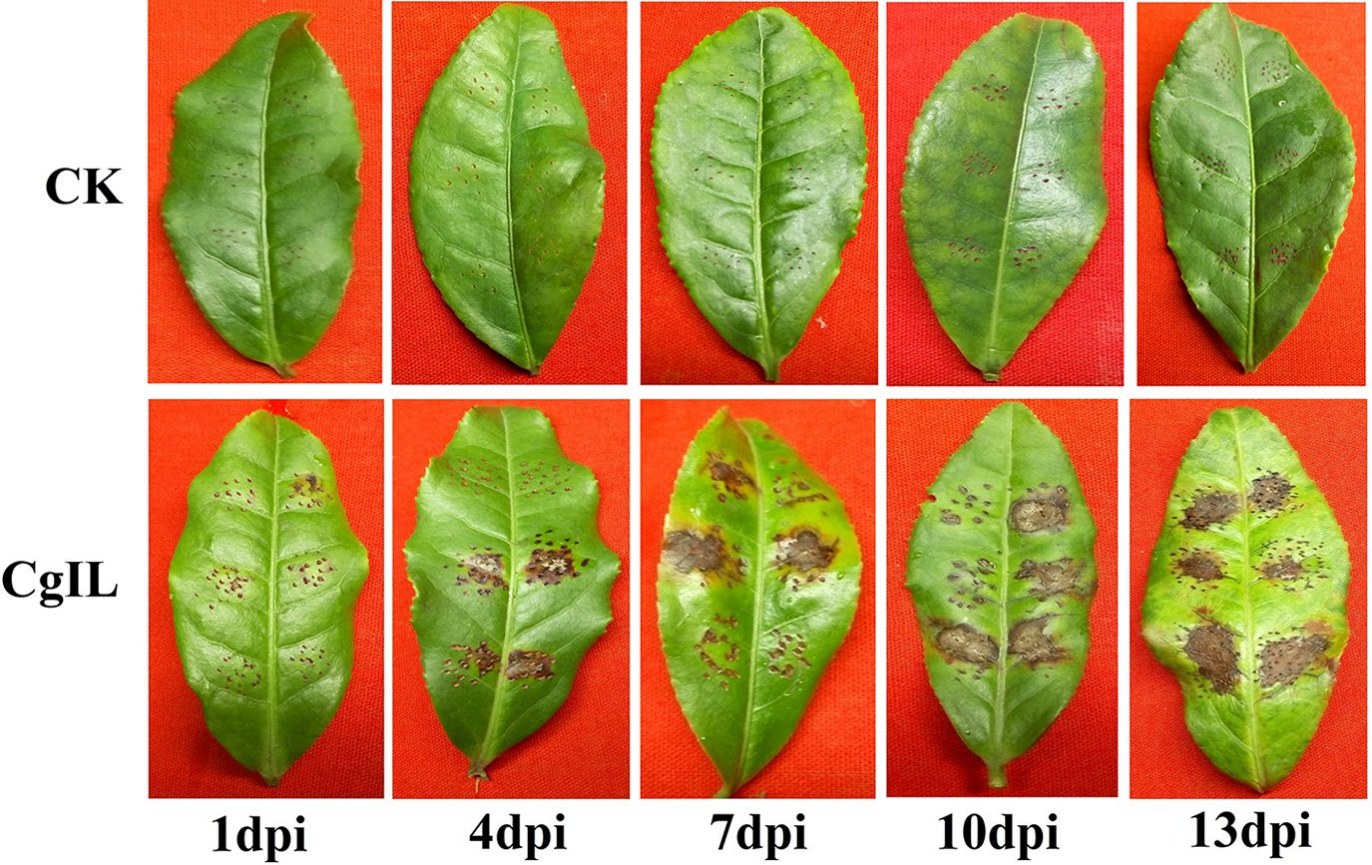
Pathogenicity test of *C. gloeosporioides* on tea plant leaves in CgIL (*C. gloeosporioides*–inoculated leaves) and CK (mock-inoculated control) after 1, 4, 7, 10, and 13 days post inoculations (dpi).

For the morphological characterization, diseased tea leaves were sliced into many pieces, surface sterilized, plated on PDA, and incubated at 28°C for 5 days. As a result, a total of seven fungal isolates were recovered from the culture plate. All the cultures on PDA exhibited white and gray color on the front side, while the back side of the colonies was of white and dark gray. For the molecular characterization, genomic DNA of the fungal isolate was amplified using ITS1 and ITS4 primers and sequenced. Based on the ITS rDNA sequence analysis, the species level identity of the fungus was confirmed as *C. gloeosporioides*, which was 100% homology with other *C. gloeosporioides* isolates (GenBank accession number: JX010223.1) (Weir et al., 2012). To identify the disease response of the cultivar of tea plant (Var.Shuchazao) against *C. gloeosporioides* infection, 3-year-old tea plant mature leaves were wounded, inoculated with conidial suspensions of *C. gloeosporioides* (10^6^ spores/ml), and then maintained in green house with high relative humidity for conidial germination. In our study, pathogenicity tests showed that the tea plant displayed the typical brown lesions of anthracnose disease around wounded areas at 4, 7, 10, and 13 days after inoculation (**Figure 1**).

### Analysis of sRNA Data From Libraries

**Figure 2:**
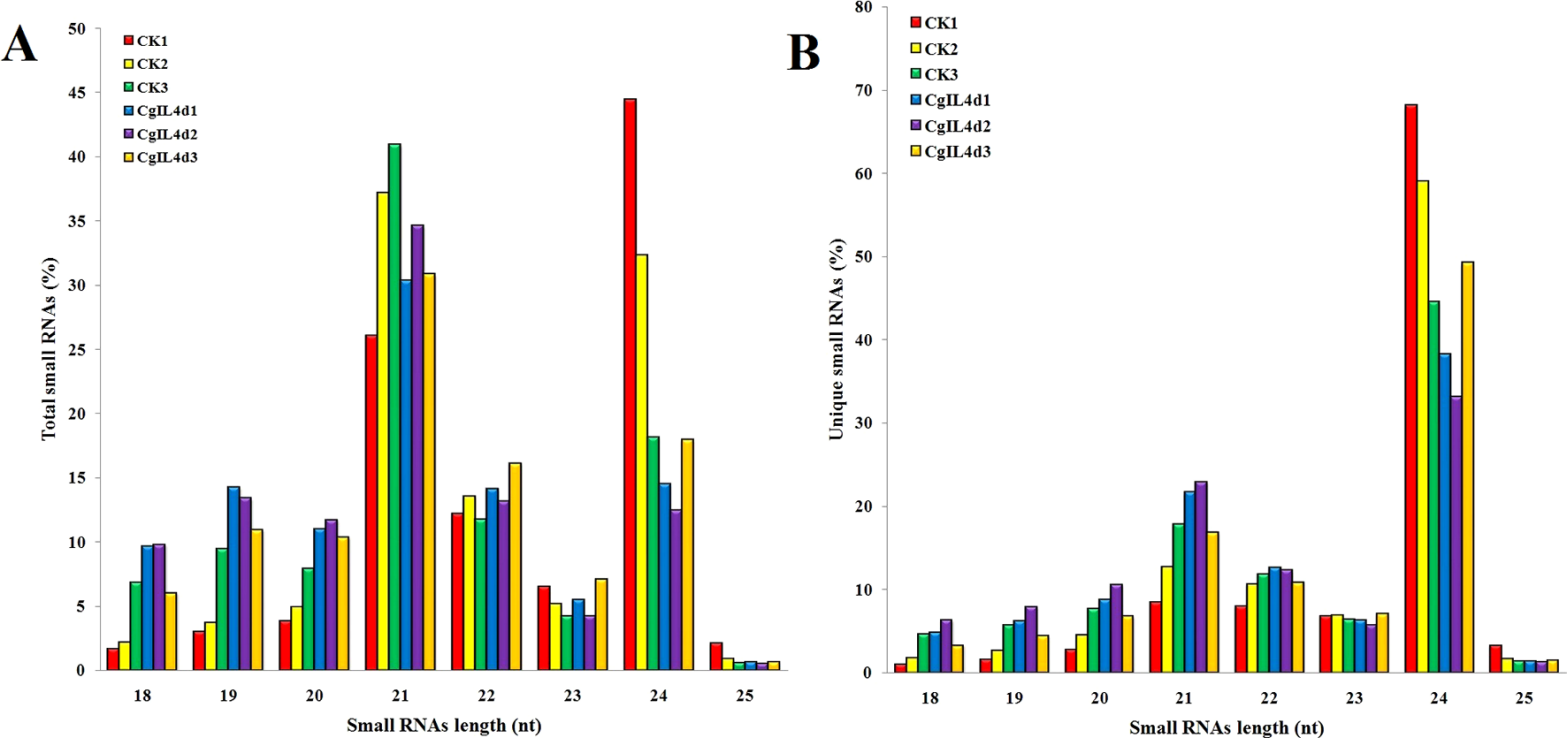
The size distributions and abundances of small RNAs. **(A)** Percentage of total small RNA and **(B)** Percentage of unique small RNA.

To identify the miRNAs in the tea plant, we sequenced sRNA in tea leaves isolated from the mock-inoculated control (CK) and the *C. gloeosporioides*-inoculated treatment (CgIL); three independent replicates of each treatment were sequenced using the Illumina GAIIX platform. A total of 66.33 million raw reads were generated, ranging from 10.37 to 12.48 million reads from each library (**Table 1**). After removing 5′ and 3′ adapter sequences, low-quality reads, and RNAs less than 18 nucleotides and longer than 25 nucleotides, the remaining reads were searched against the Rfam database (http://rfam.janelia.org), the Repbase database (http://www.girinst.org/repbase), and tea genome assembly (Wei et al., 2018). Thereafter, sRNAs were classified into different categories according to their annotations as 3′ adapter (ADT) and length filter, junk reads, Rfam, mRNA, repeats, rRNA, tRNA, snRNA, and snoRNA sequences, and other Rfam RNA sequences (**Table 1**). Finally, the total number of clean sequences was reduced to 39.70 million (**Table 1**). The number of unique sRNAs ranged from 1.49 to 4.53 million among the libraries (**Table 1**). The length distribution of sRNAs showed that the top two most abundant sRNAs were 21 and 24 nt (**Table 2** and **Figure 2**). The percentages of unique sRNAs and total sRNAs reads showed drastic differences in their ranges. Comparing reads of small RNAs between CK and CgIL libraries, the abundances of 24 nt total sRNA reads in both libraries were lower than the abundance of unique 24 nt sRNA reads, whereas the percentages of 21 nt total sRNA reads in both libraries were higher than the percentage of unique 21 nt sRNA reads; this indicated that the *C. gloeosporioides* inoculation treatment led to a significant influence on the expression pattern of small RNAs in *C. sinensis*. The abundances of 24 nt unique sRNAs ranged from 33.11% to 68.05%, whereas 21-nt unique sRNA sequences ranged from 8.52% to 22.89% in CK and CgIL libraries (**Table 2** and **Figure 2B**). In summary, 24 nt miRNAs were the most abundant, followed by 21 nt miRNA. This finding is highly consistent with those of previous studies on other tea plant cultivars (Zhang et al., 2014; Zheng et al., 2015; Liu et al., 2016) and in other plant species (Liu et al., 2015;Chen et al., 2017). However, some studies have shown 21-nt sRNAs as the most abundant in diverse plant species (Li et al., 2011; Guo et al., 2015). Thus, the 24-nt sRNAs can be important for response to *C. gloeosporioides* infection.

**Table 1:** Summary of small RNA sequences in the libraries of mock-inoculated control tea leaves at 4dpi (CK1, CK2, CK3) and *C. gloeosporioides*–inoculated infected tea leaves at 4 dpi (CgIL4d1, CgIL4d2, CgIL4d3).

Item	CK1		CK2		CK3	
	Total (%)	Unique (%)	Total (%)	Unique (%)	Total (%)	Unique (%)
Raw reads	10,957,848 (100)	5,095,079 (100)	10,372,329 (100)	4,044,624 (100)	10,469,521 (100)	3,224,037 (100)
3ADT&length filter	941,156 (8.59)	461,151 (9.05)	1,321,950 (12.74)	430,418 (10.64)	3,471,478 (33.16)	961,075 (29.81)
Junk reads	53,707 (0.49)	38,768 (0.76)	42,906 (0.41)	27,760 (0.69)	31,035 (0.3)	17,951 (0.56)
Rfam	163,216 (1.49)	8,793 (0.17)	171,980 (1.66)	8,176 (0.2)	166 201 (1.59)	8,440 (0.26)
mRNA	878,615 (8.02)	60,287 (1.18)	983,984 (9.49)	58,617 (1.45)	694,328 (6.63)	38,605 (1.2)
Repeats	2,757 (0.03)	292 (0.01)	3,879 (0.04)	346 (0.01)	4,056 (0.04)	357 (0.01)
rRNA	136,758 (1.25)	6,706 (0.06)	145,196 (1.4)	6,326 (0.06)	136,326 (1.3)	6,447 (0.06)
tRNA	9,121 (0.08)	636 (0.01)	8,320 (0.08)	514 (0)	10,725 (0.1)	672 (0.01)
snoRNA	4,213 (0.04)	412 (0)	3,707 (0.04)	355 (0)	3,359 (0.03)	322 (0)
snRNA	2,263 (0.02)	293 (0)	1,933 (0.02)	248 (0)	1,422 (0.01)	199 (0)
other Rfam RNA	10,861 (0.1)	746 (0.01)	12,824 (0.12)	733 (0.01)	14,369 (0.14)	800 (0.01)
Clean reads	8,995,659 (82.09)	4,528,076 (88.87)	7,934,269 (76.49)	3,521,692 (87.07)	6,172,590 (58.96)	2,199,975 (68.24)
Item	CgIL4d1		CgIL4d2		CgIL4d3	
	Total (%)	Unique (%)	Total (%)	Unique (%)	Total (%)	Unique (%)
Raw reads	10,734,169 (100)	2,251,034 (100)	12,478,566 (100)	2,039,551 (100)	11,327,463 (100)	2,787,299 (100)
3ADT&length filter	5,451,776 (50.79)	694,095 (30.83)	7,000,101 (56.1)	490,283 (24.04)	2,309,337 (20.39)	457,279 (16.41)
Junk reads	16,324 (0.15)	11,412 (0.51)	17,890 (0.14)	11,765 (0.58)	27,775 (0.25)	18,490 (0.66)
Rfam	172,655 (1.61)	8,306 (0.37)	280,156 (2.25)	10,181 (0.5)	296,893 (2.62)	9,887 (0.35)
mRNA	649,067 (6.05)	32,352 (1.44)	887,460 (7.11)	39,101 (1.92)	1,196,861 (10.57)	51,120 (1.83)
Repeats	2,543 (0.02)	252 (0.01)	3,471 (0.03)	311 (0.02)	3,067 (0.03)	308 (0.01)
rRNA	138,953 (1.29)	5,864 (0.05)	231,694 (1.86)	7,365 (0.06)	253,611 (2.24)	6,859 (0.06)
tRNA	17,039 (0.16)	981 (0.01)	25,444 (0.2)	1,104 (0.01)	20,171 (0.18)	1,076 (0.01)
snoRNA	6,316 (0.06)	518 (0)	7,937 (0.06)	596 (0)	10,082 (0.09)	710 (0.01)
snRNA	1,795 (0.02)	252 (0)	2,336 (0.02)	311 (0)	3,962 (0.03)	464 (0)
other Rfam RNA	8,552 (0.08)	691 (0.01)	12,745 (0.1)	805 (0.01)	9,067 (0.08)	778 (0.01)
Clean reads	4,517,428 (42.08)	1,506,720 (66.93)	4,432,594 (35.52)	1,490,805 (73.09)	7,650,496 (67.54)	2,252,700 (80.82)

**Table 2:** The length distribution and abundance of small RNAs in the libraries of mock-inoculated control tea leaves at 4 dpi (CK1, CK2, CK3) and C. *gloeosporioides*–inoculated infected tea leaves at 4 dpi (CgIL4d1, CgIL4d2, CgIL4d3).

Length	CK1		CK2		CK3	
	Total (%)	Unique (%)	Total (%)	Unique (%)	Total (%)	Unique (%)
18	149,518 (1.66)	45,378 (1)	176,549 (2.23)	61,898 (1.76)	424,682 (6.88)	102,351 (4.65)
19	272,043 (3.02)	70,449 (1.56)	294,428 (3.71)	94,231 (2.68)	583,935 (9.46)	125,690 (5.71)
20	344,648 (3.83)	127,282 (2.81)	390,503 (4.92)	159,312 (4.52)	489,998 (7.94)	169,778 (7.72)
21	2,346,628 (26.09)	385,882 (8.52)	2,947,887 (37.15)	448,355 (12.73)	2,527,051 (40.94)	391,997 (17.82)
22	1,102,384 (12.25)	360,653 (7.96)	1,075,665 (13.56)	374,799 (10.64)	727,836 (11.79)	261,189 (11.87)
23	589,500 (6.55)	308,632 (6.82)	410,870 (5.18)	244,774 (6.95)	261,291 (4.23)	140,762 (6.4)
24	3,999,383 (44.46)	3,081,291 (68.05)	2,566,526 (32.35)	2,077,119 (58.98)	1,121,863 (18.17)	977,986 (44.45)
25	191,555 (2.13)	148,509 (3.28)	71,841 (0.91)	61,204 (1.74)	35,934 (0.58)	30,222 (1.37)
Clean reads	8,995,659 (100)	4,528,076 (100)	7,934,269 (100)	3,521,692 (100)	6,172,590 (100)	2,199,975 (100)
Length	CgIL4d1		CgIL4d2		CgIL4d3	
	Total (%)	Unique (%)	Total (%)	Unique (%)	Total (%)	Unique (%)
18	435,568 (9.64)	73,006 (4.85)	434,630 (9.81)	93,833 (6.29)	462,069 (6.04)	74,286 (3.3)
19	643,790 (14.25)	93,701 (6.22)	594,451 (13.41)	117,615 (7.89)	837,351 (10.95)	100,969 (4.48)
20	496,732 (11)	132,355 (8.78)	520,136 (11.73)	157,470 (10.56)	793,105 (10.37)	152,404 (6.77)
21	1,370,503 (30.34)	325,894 (21.63)	1,534,238 (34.61)	341,239 (22.89)	2,358,603 (30.83)	378,465 (16.8)
22	637,184 (14.11)	189,646 (12.59)	583,894 (13.17)	183,113 (12.28)	1,233,251 (16.12)	244,414 (10.85)
23	249,320 (5.52)	95,674 (6.35)	189,275 (4.27)	84,809 (5.69)	543,369 (7.1)	159,761 (7.09)
24	654,980 (14.5)	575,306 (38.18)	551,668 (12.45)	493,639 (33.11)	1,372,726 (17.94)	1,108,633 (49.21)
25	29,351 (0.65)	21,138 (1.4)	24,302 (0.55)	19,087 (1.28)	50,022 (0.65)	33,768 (1.5)
Clean reads	4,517,428 (100)	1,506,720 (100)	4,432,594 (100)	1,490,805 (100)	7,650,496 (100)	2,252,700 (100)

### Identification of Conserved miRNAs

**Figure 3:**
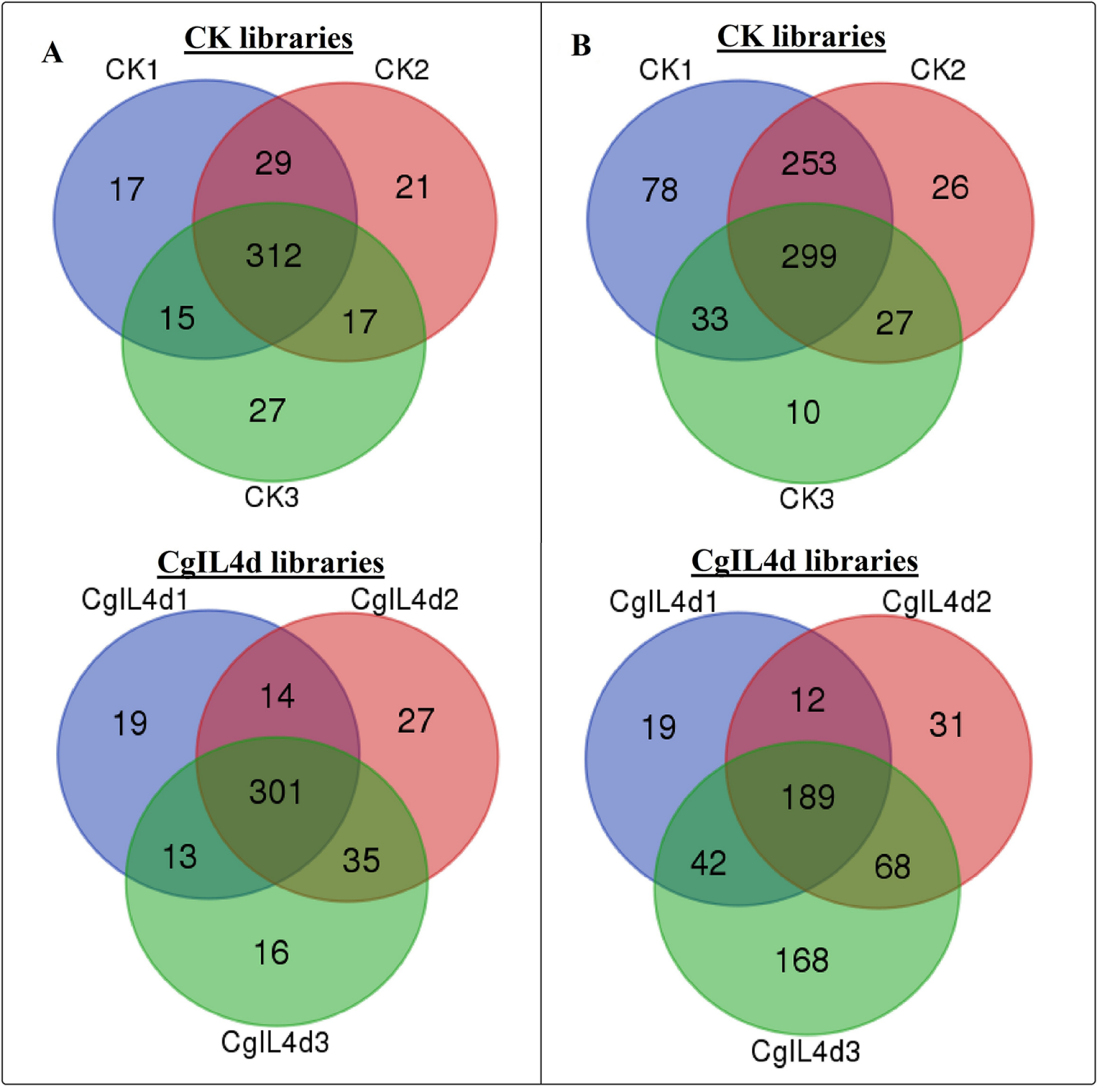
Venn diagrams showing the numbers of conserved **(A)** and novel **(B)** miRNAs identified in six samples. (A) Ser/Thr-Kinase, (B) Auxin response factor 5, (C) NAC domain-containing protein 89, (D) Nuclear transcription factor Y subunit A-3, (E) Transcription factor MYB114, (F) Calcium-dependent protein kinase.

**Figure 4:**
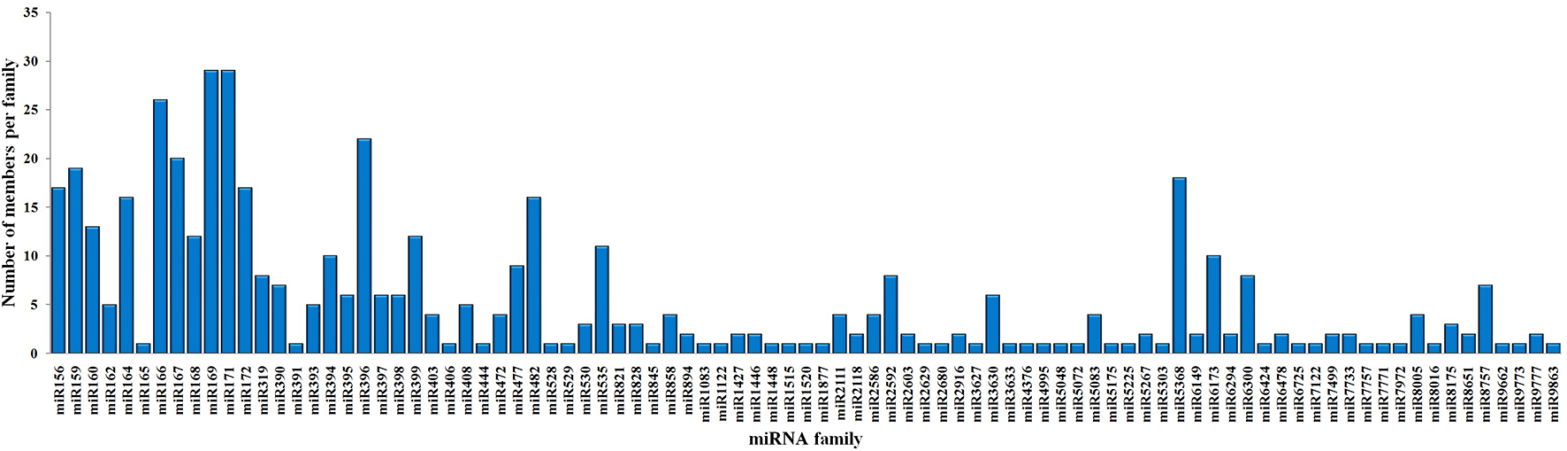
Distribution of members in conserved miRNA families in *C. sinensis*.

To identify the conserved miRNAs in tea plant, the sRNA sequences were searched against known miRNAs in miRBase 22.0 by similarity with a maximum of two mismatches and without gaps. A total of 485 conserved miRNAs were found, which corresponded to 488 pre-miRNAs belonging to 89 miRNA families (**Supplementary Table 2** and **Figure 3A**). Among these, three conserved miRNAs, csn-miR166a-3p, csn-miR166a_R+2_2, and csn-miR396b_R+1_1, showed higher abundances of reads in the CK libraries than in the CgIL4d libraries; these may play a specific role during *C. gloeosporioides* infection. Moreover, miRNAs within several conserved miRNA families, such as miR171, miR164, miR394, miR482, and miR535, had abundances ranging from 1,000 to 79,000 in at least one CK or CgIL library. The remaining miRNAs had fewer than 1,000 reads within the six libraries (**Supplementary Table 2**). Differences were found in the number of members within conserved miRNA families. Among the identified families, miR169 and miR171 families contained the most members (29), followed by miR166, miR396, and miR167 families, which had 26, 22, and 20 members, respectively. In contrast, the majority (38.20%) of miRNA families had only one member (**Figure 4**).

### Identification of Novel miRNAs

To identify potentially novel miRNAs, reads that did not match any known miRNAs were further analyzed. All un-annotated unique sRNA sequences were mapped to the scaffold sequences of genome assembly. The stem-loop secondary structure of miRNA precursors was predicted using the mfold software (http://mfold.rna.albany.edu/?q=mfold/RNA-Folding-Form) (Zuker, 2003). According to aforementioned criteria, we identified 761 novel miRNAs corresponding to 488 pre-miRNAs in all six libraries (**Supplementary Table 3** and **Figure 3B**). The abundances of four novel miRNAs were significantly different between the CK and CgIL libraries; in addition, among 761 miRNAs, 726 and 529 miRNAs were only detected in the CK and CgIL libraries, respectively (**Supplementary Table 3**). These findings indicate that levels of certain novel miRNAs are specifically repressed by *C. gloeosporioides* stress. The PC-3p-29_74070, PC-3p-23_113175, PC-3p-69_25219, and PC-3p-721_2531 were the most abundant miRNA classes with more than 1,000 reads, and the remaining miRNAs had fewer than 1,000 reads in all six libraries (**Supplementary Table 3**). The sequences of novel miRNAs were 19 to 25 nt in length, and 24 nt reads were the most abundant among the six libraries, followed by 21, 22, 20, 23, 19, and 25 nt. The lengths of novel miRNA precursors ranged from 51 to 220 nt, with an average length of 136 nt. The negative folding free energies of the novel miRNA precursors ranged from –187.6 to –18.1 kcal mol^-1^, with an average value of –102.85 kcal mol^-1^. The folding structures of the predicted novel miRNAs are shown in **Supplementary Table 3**.

### Differential Expression Analysis of miRNAs

**Figure 5:**
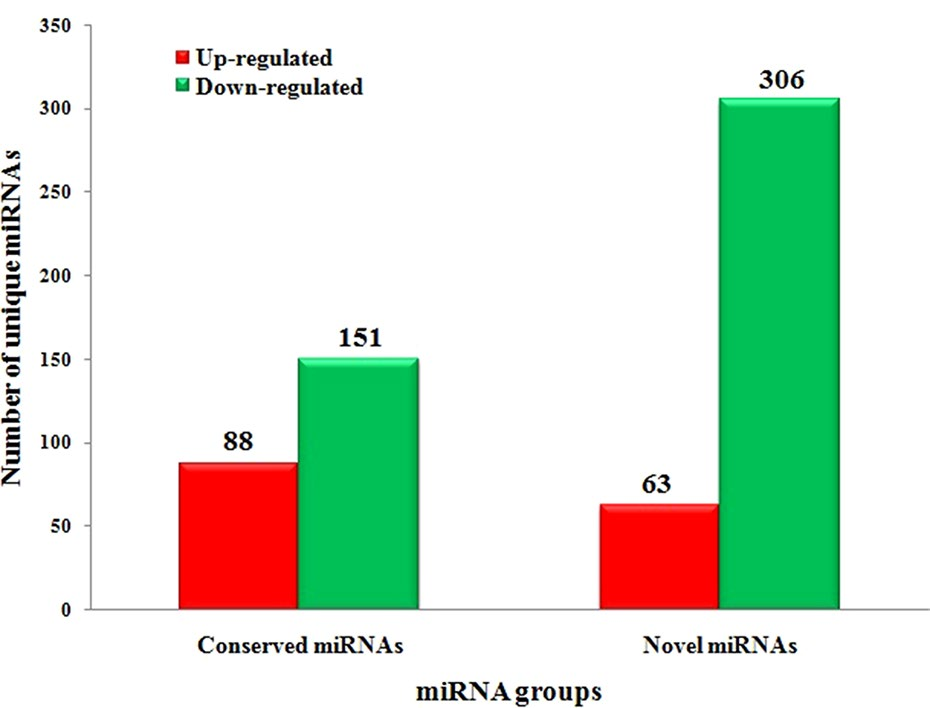
Significantly differentially expressed conserved and novel miRNAs between CgIL4d vs. CK.

To systematically identify *C. gloeosporioides* stress responsive miRNAs in tea plant, we identified DEMs with at least twofold change in abundance (*p* < 0.05) (**Supplementary Table 4** and **Figure 5**). In total, 88 conserved miRNAs were significantly up-regulated and 151 conserved miRNAs were significantly down-regulated in the CgIL libraries compared with the CK libraries. Of these miRNAs, csn-miR398a was the most significantly up-regulated miRNA (7.65-fold), while csn-miR171j-5p_2ss8CT9TC was the most significantly down-regulated miRNA (−6.28-fold) (**Supplementary Table 4** and **Figure 5**). We also identified 63 up-regulated novel miRNAs and 306 down-regulated novel miRNAs that showed significant differential expression between the CgIL and CK libraries. Of these miRNAs, the most up-regulated miRNA was PC-5p-6208_284 (3.43-fold), while the most down-regulated miRNA was PC-3p-3475_476 (−7.63-fold) (**Supplementary Table 5** and **Figure 5**). Two hundred thirty-six miRNAs (59 conserved and 177 novel miRNAs) were unique to the CK libraries, whereas 115 miRNAs (48 conserved and 67 novel miRNAs) were unique to the CgIL libraries; thus, these differentially expressed miRNAs might play crucial roles in response to *C. gloeosporioides* stress in tea plant.

### Identification of miRNA Targets

To explore the potential miRNA targets and their biological functions during *C. gloeosporioides* stress in tea plant, we performed genome-wide analysis of miRNA-cleaved mRNAs using high-throughput degradome sequencing technology (Addo-Quaye et al., 2008; Gao et al., 2004). A total of 18,056,656 and 14,212,790 reads were identified in the CK and CgIL libraries, respectively (**Table 3**); of these reads, 74.89% and 77.38% could be mapped to the *C. sinensis* genomic assembly, respectively (**Table 3**). Using the CleaveLand3.0 tool, a total of 1,134 conserved and 596 potentially novel miRNA targets were predicted in the degradomes of CK and CgIL, respectively. The abundances of targets were classified in accordance with previous criteria and are shown in **Supplementary Tables 6** and **7** (Li et al., 2010; Addo-Quaye et al., 2008). For CK targets, 205 (13.85%), 19 (1.28%), 551 (37.23%), 71 (4.80%), and 634 (42.84%) were grouped into categories 0, 1, 2, 3, and 4, respectively; meanwhile, for the CgIL targets, 125 (16.13%), 10 (1.29%), 313 (40.39%), 41 (5.29%), and 286 (36.90%) were grouped into categories 0, 1, 2, 3, and 4, respectively (**Supplementary Tables 6** and **7**). We further examined the abundance changes of targets with the treatment, which revealed that 1,487 and 775 targets had significantly changed sites with more than twofold change (*p* < 0.05) in CK and CgIL, respectively (**Supplementary Tables 6** and **7**). These results indicate that most of the predicted targets are efficiently cleaved by their corresponding miRNAs.

**Table 3:** Summary of degradome library data.

Library		Raw reads	Unique raw reads	cDNA mapped reads	Unique cDNA mapped reads	Number of input cDNAs	Number of covered cDNAs
CK (%)	Number	18,056,656	7,027,557	13,522,760	4,862,196	163,291	76,236
	Ratio	*–*	*–*	74.89 %	69.19 %	*–*	46.69 %
CgIL (%)	Number	14,212,790	4,314,069	10,998,394	2,953,113	163,291	61,860
	Ratio	*–*	*–*	77.38 %	68.45 %	*–*	37.88 %

CK, mock-inoculated leaves; CgIL, C.gloeosporioides-inoculated leaves.

In the present study, 1,134 conserved and 596 potentially novel miRNA targets were predicted based on their perfect or near-perfect complementarity to their target gene sequences in the degradomes of CK and CgIL. Of these, 311 and 823 target transcripts were up- and down-regulated by 137 (82 up-regulated and 55 down-regulated miRNAs) and 230 (100 up-regulated and 130 down-regulated miRNAs) differentially expressed conserved miRNAs, respectively, while 131 and 465 targets were potentially up- and down-regulated by 79 (33 up-regulated and 46 down-regulated miRNAs) and 216 (67 up-regulated and 149 down-regulated miRNAs) differentially expressed novel miRNAs, respectively. We defined a predicted miRNA target gene when its expression pattern was in contrast with that of the miRNA, reflecting the fact that mRNA expression was negatively correlated with miRNA expression. Based on these rules, our results indicate that most of the predicted targets of conserved and novel miRNAs are highly negatively correlated with their corresponding miRNAs. These negative correlations positively regulate various metabolic, biological, and cellular processes.

### Functional Analysis of miRNA Targets

**Figure 6:**
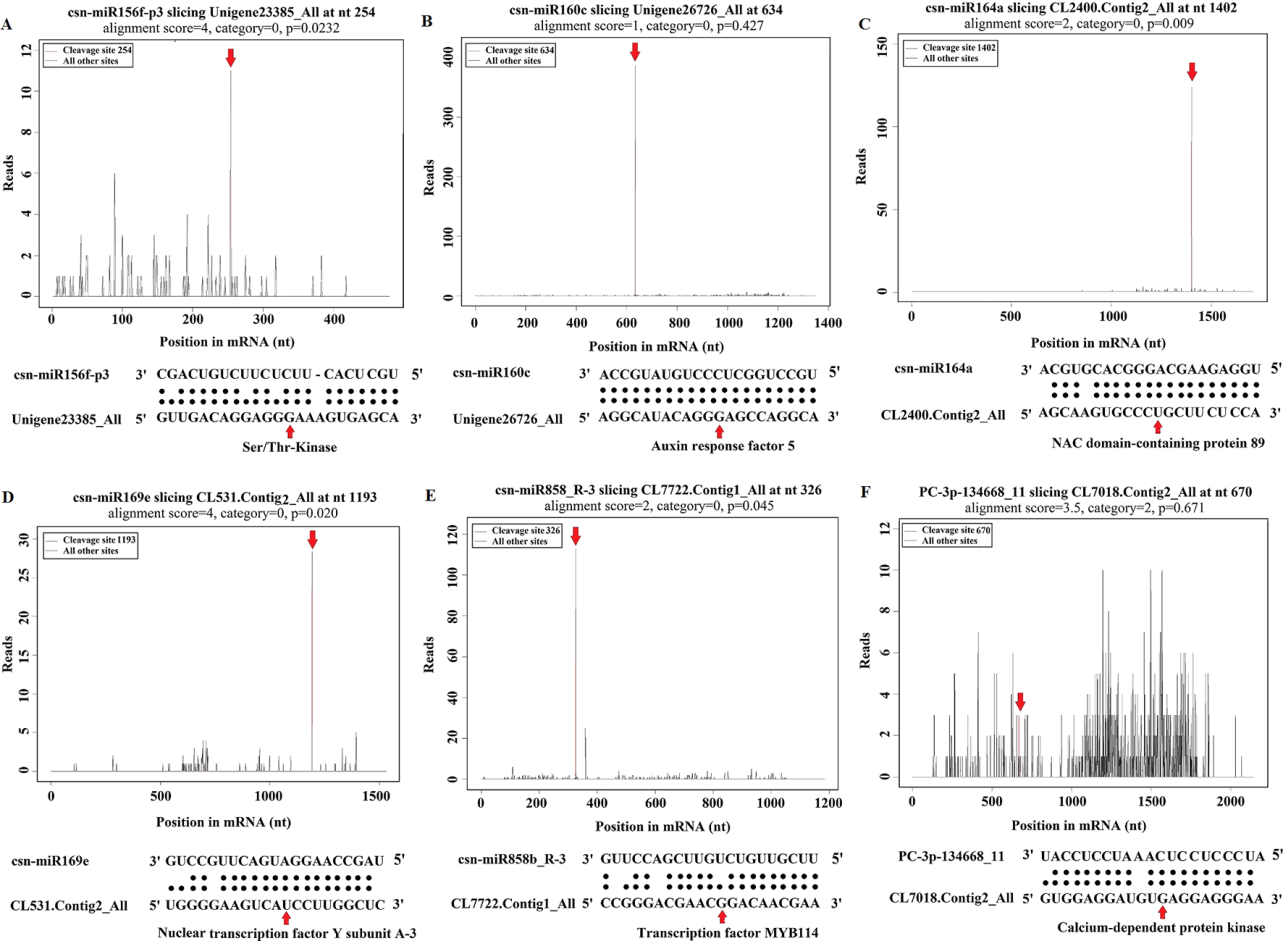
Target plot (t-plot) of representative tea miRNA targets mined from degradome sequencing. **(A)** Ser/Thr-Kinase, **(B)** Auxin response factor 5, **(C)** NAC domain-containing protein 89, **(D)** Nuclear transcription factor Y subunit A-3, **(E)** Transcription factor MYB114, **(F)** Calcium-dependent protein kinase.

Gene functional annotation analysis showed that the identified targets include transcription factors, such as auxin response factor (ARF), Myb domain proteins (MYBs), NAC domain transcription factors (NACs), nuclear transcription factor Y (NFYs), and WRKY family transcription factor (WRKYs), which regulate plant growth and development as well as stress responses (**Figure 6**, **Supplementary Tables 6** and **7**). Most of the target genes were protein-coding genes, including serine/threonine-protein kinase (Ser/Thr_kinase), leucine-rich repeat protein kinases (LRR-RLKs), calcium-dependent protein kinase (CDPK), mitogen-activated protein kinase (MAPK), and auxin-responsive protein, which are involved in signal sensing and transduction (**Figure 6**, **Supplementary Tables 6** and **7**). In addition, we identified several target genes related to plant defense, including L-ascorbate oxidase, cytochrome C oxidase, beta-glucosidase, glutathione synthetase, glutathione S-transferase, glutathione reductase, catalase, peroxidase, phenylalanine ammonia-lyase (PAL), and cinnamoyl-CoA reductase (CCR). The other predicted target genes like Calmodulin (CaM) like proteins and ROS appeared to be involved in diverse physiological and metabolic processes, such as plant metabolism, transport, and stress responses (**Supplementary Tables 6** and **7**). In our study, we found that csn-miR394b_L-1_1ss5CA was significantly down-regulated, and its targeted gene encodes CaM like proteins, which may cause influx of calcium after *C. gloeosporioides* infection in tea plant. In addition, csn-miR164a-p3/p5_2ss15CG18CG also targets CaM like proteins (**Supplementary Table 6**). Consistent with our results, Nischal et al. (2012) showed that miR164 inversely regulates the calmodulin-related calcium sensor protein in rice under abiotic stress. Calcium influx is triggered immediately by PAMP perception (Ranf et al., 2011). The extracellular calcium signals generated after pathogen attacks are transmitted to calmodulins (CaM) and calmodulin-binding proteins (CBP), which receive signals from the calmodulins, activate CDPKs, and trigger transcription of specific defense genes (Dodd et al., 2010). Studies have shown that overexpression of CBP in Arabidopsis causes elevated SA accumulation, increased expression of the defense genes, and enhanced resistance to *Pseudomonas syringae* (Wan et al., 2012). Our findings indicate that these miRNAs are likely involved in calcium activated SA biosynthesis in tea plant. We observed that CDPKs were targeted by two up-regulated miRNAs (csn-miR482b-p5_2ss3TA19T and PC-3p-134668_11) and one down-regulated miRNA (csn-miR169e_R-3) after *C. gloeosporioides* infection in *C. sinensis* (**Supplementary Tables 6** and **7**). This result suggested that these miRNAs are involved in protein kinase induced Ca^2+^ signal events, thereby triggering miRNA-mediated regulations.

**Figure 7:**
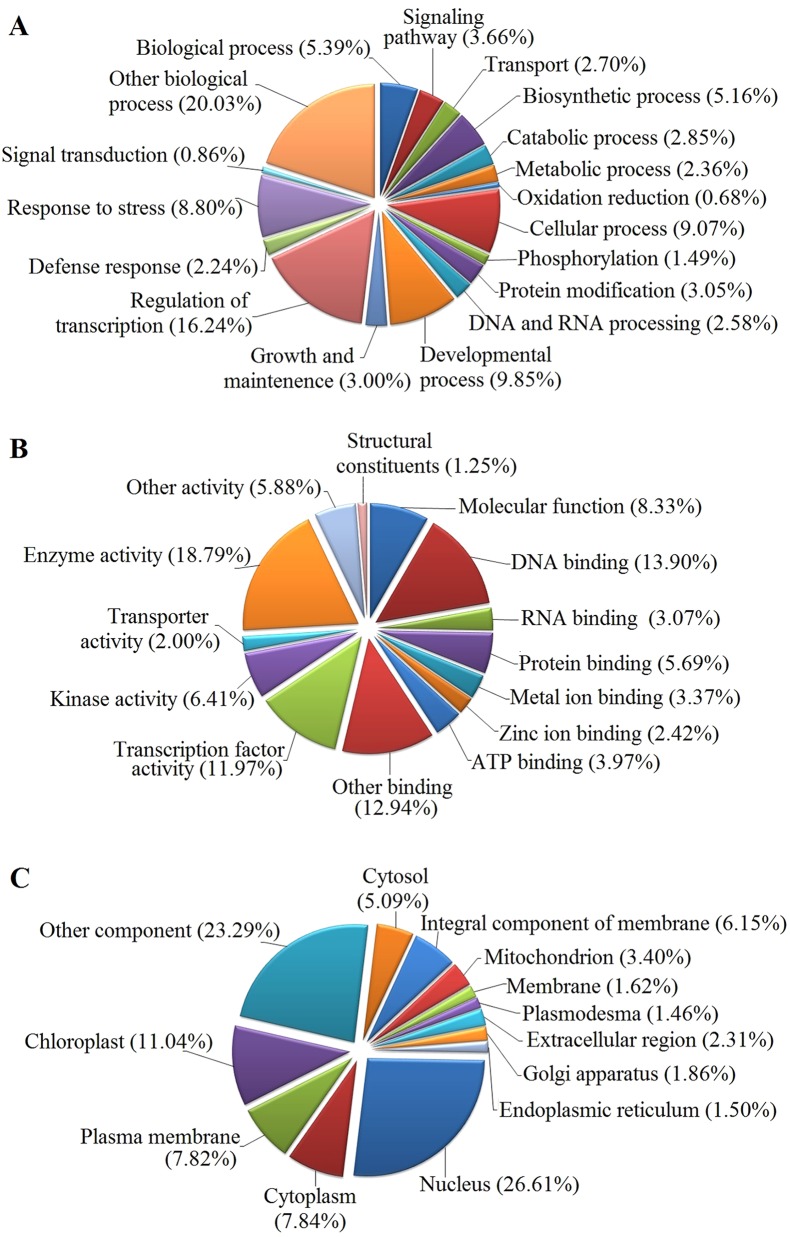
GO analysis of predicted putative target transcripts of all miRNAs. Categorization of miRNA target transcripts was performed according to biological process **(A)**, molecular function **(B)**, and cellular component **(C)**.

**Figure 8:**
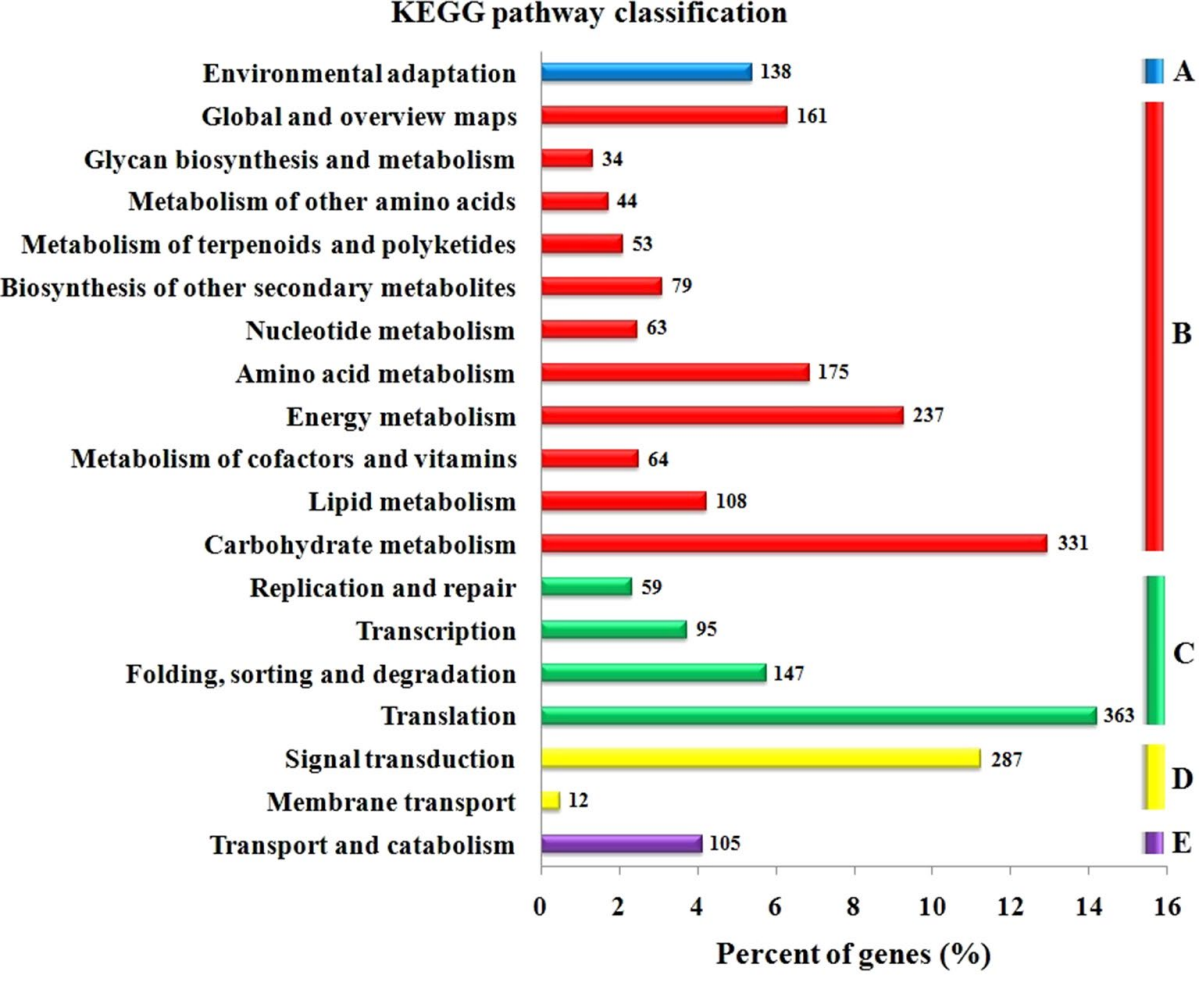
KEGG pathway classification for predicted putative target transcripts of miRNAs between CgIL and CK. The KEGG pathways were categorized into five groups. **(A)** Organismal system, **(B)** metabolism, **(C)** genetic information processing, **(D)** environmental information processing, and **(E)** cellular process.

The GO functional annotation of all predicted miRNA targets were conducted using Blast2GO program (**Supplementary Table 8** and **Figures 7A–C**). In the GO biological process category, the predominant terms were regulation of transcription (16.24%), response to stress (8.80%), biosynthetic processes (5.16%), and signaling pathway (3.66%) (**Figure 7A**). In the GO molecular function category, the most frequent term was enzyme activity (18.79%), followed by DNA binding (13.90%), transcription factor activity (11.97), and kinase activity (6.41) (**Figure 7B**). With regard to the GO cellular component category, nucleus, chloroplast, plasma membrane, and cytoplasm were the most frequent terms (**Figure 7C**). Moreover, a total of 122 KEGG pathways were enriched for the target genes of the 276 conserved and 253 novel differentially expressed miRNAs. Target genes within enriched KEGG pathways were mainly involved in 19 pathways. Among them, translation (14.20%), carbohydrate metabolism (12.95%), signal transduction (11.23%), energy metabolism (9.28%), and amino acid metabolism (6.85%) were the five major pathways. Furthermore, all the KEGG pathways were categorized into five groups including organismal system (5.40%), metabolism (52.80%), genetic information processing (25.99%), environmental information processing (11.70%) and cellular process (4.11%) (**Supplementary Table 9** and **Figure 8**).

### Expression Analysis of miRNAs and Their Targets

**Figure 9:**
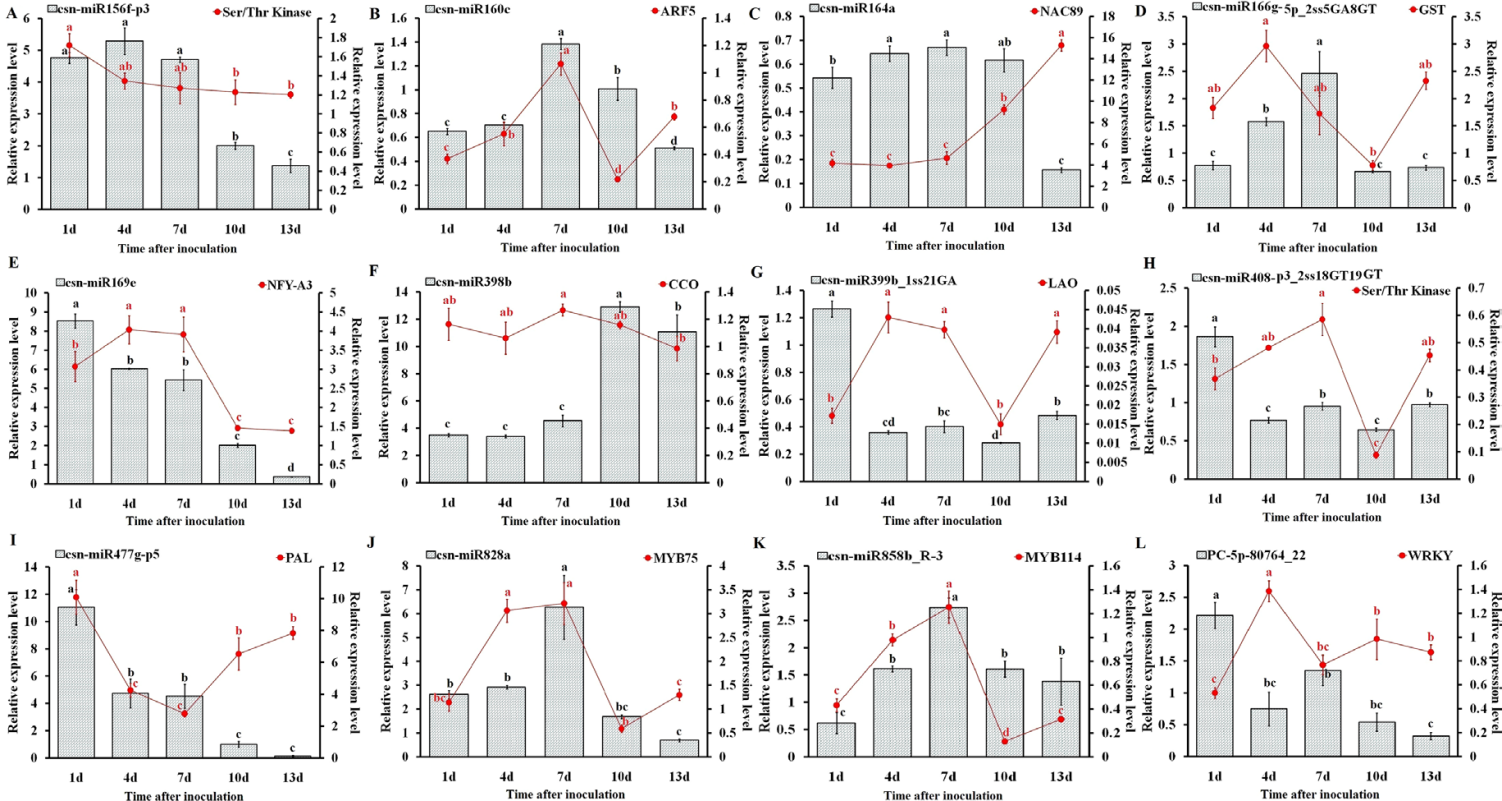
Expression analysis of miRNAs and target genes (A–L). The bars and lines represent the abundances of miRNAs and their corresponding targets in *C. sinensis* leaves exposed to *C. gloeosporioides* at different time intervals by qRT-PCR: csn-miR156f-p3 **(A)**, csn-miR160c **(B)**, csn-miR164a **(C)**, csn-miR166g-5p_ 2ss5GA8GT **(D)**, csn-miR169e **(E)**, csn-miR398b **(F)**, csn-miR399b_1ss21GA **(G)**, csn-miR408-p3_2ss18GT19GT **(H)**, csn-miR477g-p5 **(I)**, csn-miR828a **(J)**, csn-miR858b_R-3 **(K)**, and PC-5p-80764_22 **(L)**. The Y-axis on the left and right indicate the expression levels of the miRNAs and targets, respectively. U6 SnRNA and GADPH were used as an internal control for miRNAs and targets, respectively. The expression levels of the miRNAs and targets in the control tea plant leaves at different time points (1, 4, 7, 10, and 13 dpi) were set as 1.0. Relative expression was calculated using the 2^-∆∆CT^ method. Data represent the mean ± SD values of three biological replicates. Different letters above the bars represent significant differences at *p* < 0.05. Means followed by the same letter over the bars are not significantly different at the 0.5% level according to DMRT analysis.

To understand the physiological importance and the regulatory mechanisms of miRNAs in tea plant, the expression pattern of 12 randomly selected miRNAs and their target genes at different *C. gloeosporioides* inoculation time points (1, 4, 7, 10, and 13 days) was determined through qRT-PCR analysis (**Figure 9**). As shown in **Figure 9**, the qRT-PCR findings showed that the expression pattern of five miRNAs, csn-miR160c, csn-miR164a, csn-miR166g-5p_2ss5GA8GT, csn-miR828a, and csn-miR858b_R-3, were significantly higher at 7 dpi, while other five miRNAs, csn-miR169e, csn-miR399b_1ss21GA, csn-miR408-p3_2ss18GT19GT, csn-miR477g-p5, and PC-5p-80764_22, showed higher expression at 1 dpi. In addition, other two miRNAs, csn-miR156f-p3 and csn-miR398b, exhibited increased levels of expression at 4 and 10 dpi, respectively. On the other hand, the expression levels of eight miRNAs (csn-miR156f-p3, csn-miR160c, csn-miR164a, csn-miR166g-5p_2ss5GA8GT, csn-miR169e, csn-miR477g-p5, csn-miR828a, and PC-5p-80764_22) were significantly decreased in CgIL leaves at 13 dpi relative to the mock-inoculated CK control leaves. The opposite expression pattern between miRNAs and corresponding target transcripts were observed in CgIL leaves at different time intervals. For example, the expression levels of csn-miR160c, csn-miR164a, csn-miR477g-p5, csn-miR828a, and csn-miR858b_R-3 in CgIL leaves were decreased, while the expression levels of corresponding target transcript (ARF5, NAC89, PAL, MYB75, and WRKY) were increased at 10 to 13 dpi. In addition, the expression levels of csn-miR169e, csn-miR399b_1ss21GA, and csn-miR408-p3_2ss18GT19GT were significantly decreased in CgIL leaves relative to the control, while the increased expression was observed for their corresponding target transcripts (NFY-A3, LAO and Ser/Thr Kinase) at 1 to 4 dpi.

### Correlation Analysis Between miRNA and Target Gene Expression

We have correlated the differential expression of 12 randomly selected miRNAs and their target genes. We found that csn-miR164a, csn-miR169e, csn-miR477g-p5, csn-miR828, csn-miR858b, and PC-5p-80764_22 were negatively correlated with their respective targets. The remaining six miRNAs partially showed positive correlation with their target genes. All the related target genes showing non-additive expression were also significantly enriched in “response to stress” and response to “biotic” in the present study, but negatively correlated gene targets were significantly enriched in the “metabolic process” category. We have also correlated the qRT-PCR expression of these 12 randomly selected miRNA-target genes. Interestingly, the expression of novel miRNA PC-5p-80764_22 exhibited a negative correlation with its target gene WRKY in tea plant samples throughout the entire period of infection (1 to 13 dpi). This shows that this novel miRNA regulates the WRKY gene throughout the infection. At 7 dpi, most of the miRNAs showed negative correlation with their respective targets. However, in general, the correlation between the expression of remaining 11 miRNAs and their respective target genes varied depending on the time points, indicating that additional factors may be involved in regulating these target genes and that miRNA-target gene interactions are extremely complex (**Table 4**).

**Table 4:** Correlations between the expression of 12 tea miRNAs and their target genes during *C. gloeosporioides* infection.

miRNA/Target	Leaf (r values)				
	1 dpi	4 dpi	7 dpi	10 dpi	13 dpi
csn-miR156f-p3/ STK1	**0.94**	**0.88**	-0.63	**0.99**	**0.46**
csn-miR160c/ARF	**0.50**	-0.97	-0.68	-0.58	-0.31
csn-miR164a/NAC	-0.41	-0.21	**0.92**	-0.27	-0.29
csn-miR166g-5p/GST	**0.95**	**0.90**	-0.04	**0.85**	**0.24**
csn-miR169e/NFY	-0.89	**0.49**	**0.92**	-0.24	-0.89
csn-miR398b/Cox	**0.61**	-0.81	-0.58	**0.62**	-0.25
csn-miR399b/LAO	**0.50**	**0.82**	-0.39	**0.47**	-0.53
csn-miR408-p3/STK2	-0.97	**0.70**	-0.36	-0.22	**0.99**
csn-miR477g-p5/PAL	-0.99	**0.44**	**1.00**	-0.92	**0.84**
csn-miR828a/MYB75	**0.95**	**0.71**	-0.49	-0.99	-0.87
csn-miR858b_R-3/MYB114	**0.57**	**0.61**	-0.33	**0.94**	**0.91**
PC-5p-80764_22/WRKY	-0.95	-0.94	-0.96	-0.68	-0.92

“1 dpi, 4 dpi, 7 dpi, 10 dpi, 13 dpi” indicate the number of days post inoculation; “values bolded with +“ indicate positive correlation, “values with -“ indicate negative correlation; “r” indicates correlation coefficient values. Results of expression correlation between miRNA and its target genes during CgIL infection were analyzed by Pearson’s correlation analysis.

### Experimental Verification of miRNA-Guided Cleavage of Target mRNAs in Tea Plant

**Figure 10:**
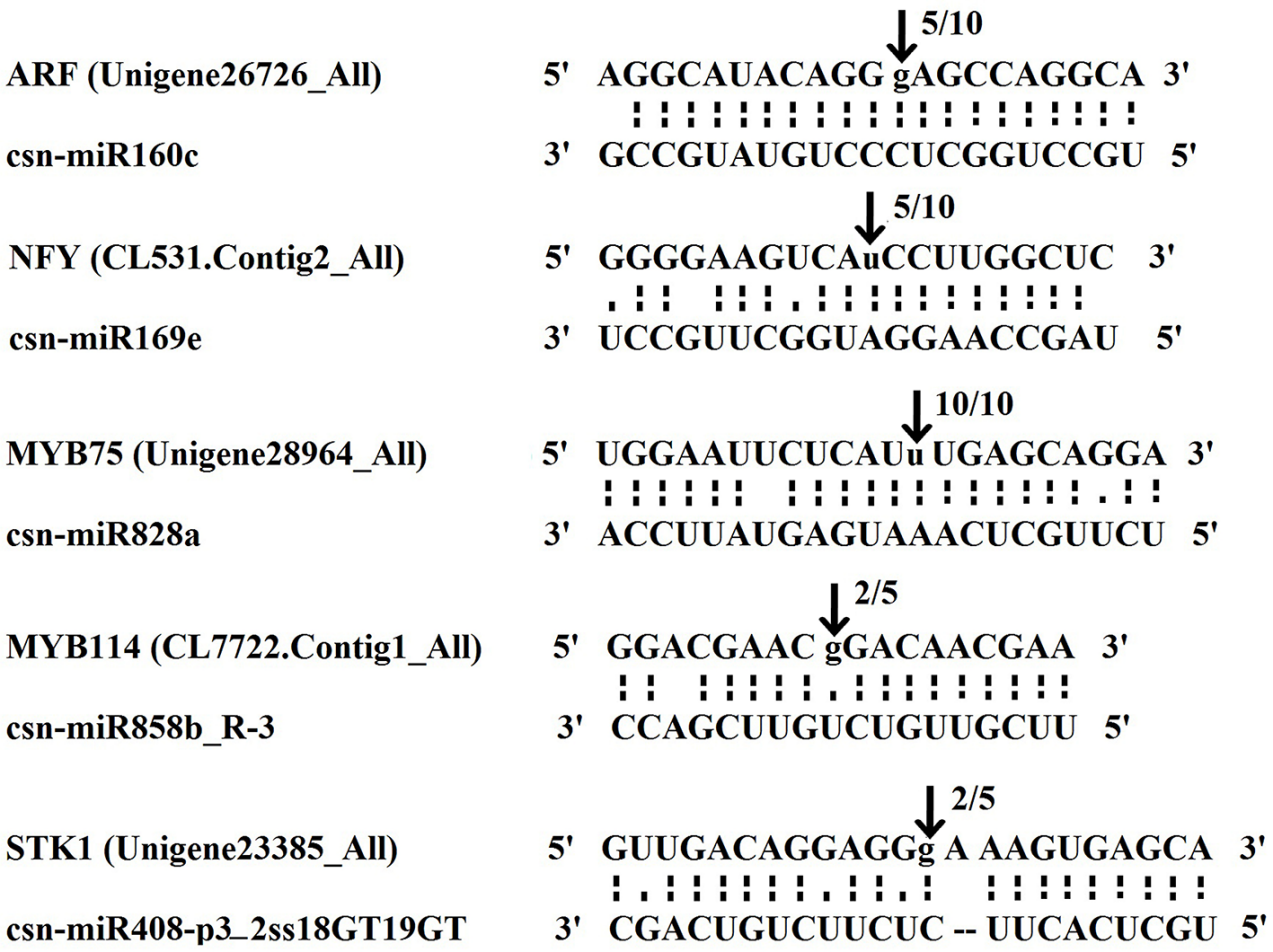
The mRNA cleavage sites of miRNAs identified by 5’ RLM-RACE. Each top strand depicts a miRNA complementary site, and each bottom strand depicts the anti-parallel miRNA. The arrows indicate the 5’ termini of mRNA fragments isolated from tea plant, as identified by the RLM-RACE product, with the frequency of clones shown above. The numbers indicate the fraction of cloned PCR products terminating at different positions.

To examine the predicted targets of miRNAs, we used 5’RLM-RACE to validate the cleavage sites in the target mRNA. All the 5’RLM-RACE PCR products amplified from predicted targets of five conserved miRNAs were analyzed on agarose gels, purified, cloned, and sequenced. The sequencing results revealed that the cleavage site of Ser/Thr-protein kinase (Unigene23385_All) and MYB75 (Unigene28964_All) lies between the 12^th^ and 13^th^ bases from the 5’ end pairing of csn-miR408-p3_2ss18GT19GT and csn-miR828a, respectively. The cleavage site of ARF (Unigene26726_All) lies between the 11^th^ and 12^th^ bases from the 5’ end pairing of csn-miR160c. NFY (CL531.Contig2_All) and MYB114 (CL7722.Contig1_All) were verified as targets of csn-miR169e and csn-miR858b_R-3, respectively. NFY can be regulated by cleavage in the binding region between the 10^th^ and 11^th^ bases from the 5’ end pairing of csn-miR169e, and MYB114 can be regulated by cleavage in the binding region between the 8^th^ and 9^th^ bases from the 5’ end pairing of csn-miR858b_R-3 (**Figure 10**). These validated sites were consistent with the predicted sites.

### Hypothetical Model of miRNA-Mediated Regulation

**Figure 11:**
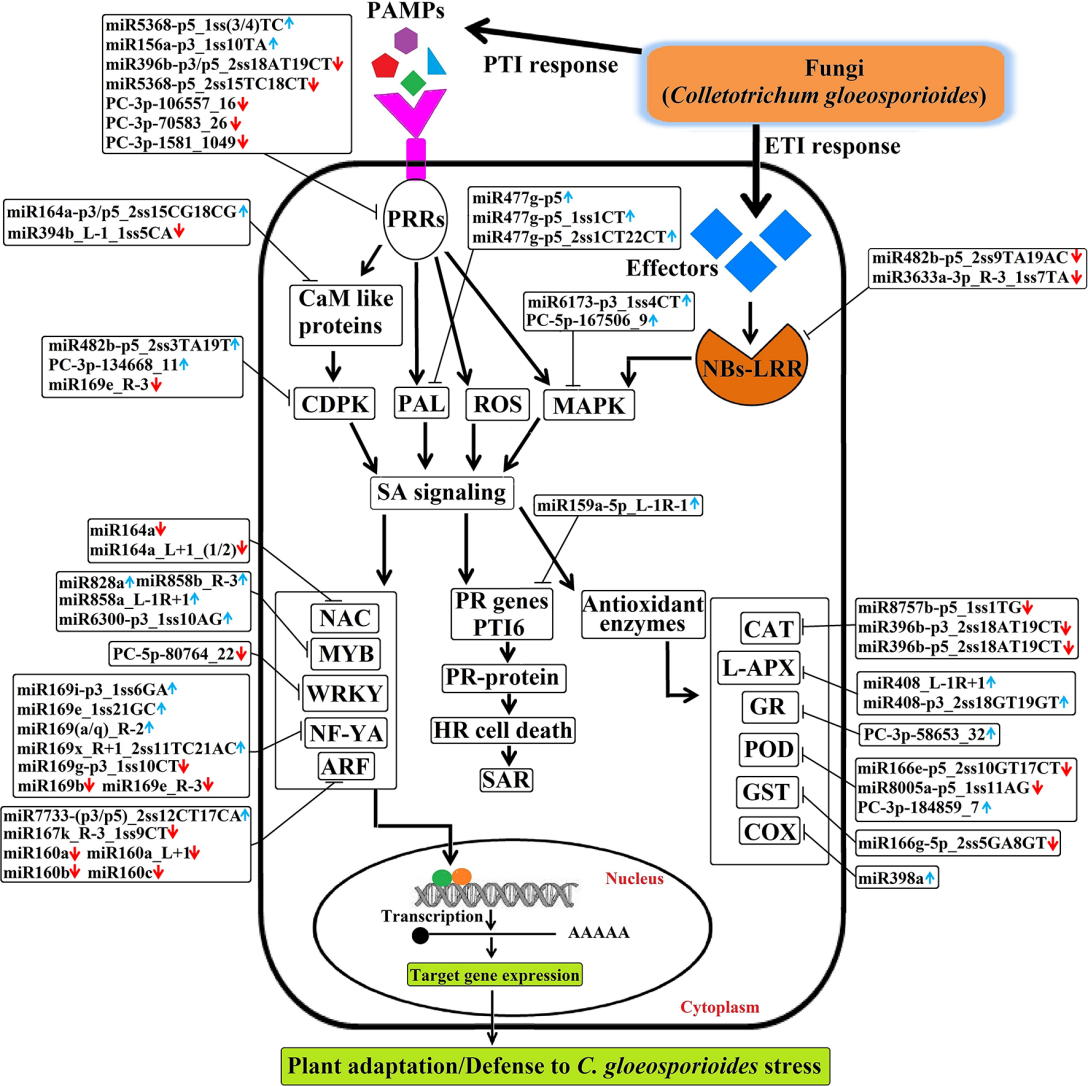
The hypothetical model of regulatory networks of *Colletotrichum gloeosporioides*–responsive miRNAs and their target genes in tea plant.

Briefly, the regulatory network, functional sharing of putative target genes, and expression profile data were used to construct a hypothetical model that illustrates how target genes influence *C. gloeosporioides* pathosystem at the biological level (**Figure 11**). Many conserved and novel miRNAs found in this study were associated with responses to various abiotic and biotic stresses, respectively. In this order, target genes of eight conserved miRNAs (csn-miR8757b, csn-miR396b, csn-miR408, csn-miR398a, csn-miR166e, csn-miR8005a, csn-miR821b, and csn-miR477) and three novel miRNAs (PC-3p-58653_32, PC-3p-180347_8, and PC-3p-184859_7) were confirmed to regulate various antioxidant genes and intensely induced H_2_O_2_. All these miRNAs may correlate with pathways associated with reactive oxygen species (ROS) and play important roles in stress responses mediated by glycosylating hormones and secondary metabolites. Apart from this, target genes of miR393, miR160, and miR167 are known to regulate downstream gene expression of auxin pathway *via* regulating transport inhibitor response 1 (TIR1) and ARF in response to stress factors and components of stress signal transduction pathways. In contrast, transcription factor genes MYB, NAC, and NF-YA controlled by miRNAs (csn-miR828a, csn-miR858b, csn-miR858a, csn-miR6300, csn-miR164a, and csn-miR169) are identified to be a positive regulator of abiotic and biotic stress response. Most conserved signaling cascade pathway (MAPKs) linked with disease resistance *via* SA signaling pathway was mainly targeted by csn-miR6173 and PC-5p-167506_9. In addition, csn-miR159a targeted a transcriptional activator of pathogenesis-related (PR) genes (PTI6), which controls the SA-mediated pathway associated with programmed cell death during the hypersensitivity response. The target genes of three conserved miRNAs such as miR396, miR5368, and miR156a and four novel miRNA, namely PC-5p-80764_22, PC-3p-1581_1049, PC-3p-106557_16, and PC-3p-70583_26, may belong to WRKY transcription factor, LRR protein kinase, Ser/Thr-kinase, and LRR receptor, which are well-known to play important roles in defense responses and disease resistance in plant–pathogen interactions. The target genes of csn-miR164a and csn-miR168 are a beta-glucosidase that triggers a plant-defense-related product in response to fungal pathogens. csn-miR394 and csn-miR164a target genes encode CaM like proteins, which receive signals from the calmodulins and lead to calcium activated SA biosynthesis through CDPKs and trigger transcription of specific defense genes (Dodd et al., 2010). The hypothetical model reveals that association of miRNAs and target genes can influence different metabolic pathways and cellular processes, which leads to various biological responses. All these mutual effects allow tea plant to be susceptible with *C. gloeosporioides* infection-related stress at the biological level (**Figure 11**).

## Discussion

During plant fungal infection, miRNAs are thought to be involved in both PTI and ETI mechanisms to regulate various gene expressions (Weiberg et al., 2014). Anthracnose disease caused by *C. gloeosporioides* is one of the most destructive fungal diseases in tea plant (Fang et al., 2013). Little is known about the roles of miRNAs in tea plant under biotic stress of *C. gloeosporioides* infection, although miRNAs and their target genes have been identified in tea plant during abiotic stresses such as cold and drought (Zhang et al., 2014; Liu et al., 2016). Here, we identified *C. gloeosporioides*-responsive miRNAs and investigated their target mRNAs using high throughput sequencing in tea plant.

We identified miRNAs consisting of 485 conserved and 761 novel miRNAs in this study. Among these, some of the conserved miRNA families are of interest and have been reported to be involved in biotic stress responses. For example, miR159, miR164, miR166, miR168, miR171, miR394, miR396, miR482, and miR535 showed higher abundances, while other miRNA families (miR156, miR160, miR169, miR319, miR393, miR395, miR396, and miR408) showed lower abundances after CgIL treatment. Previous reports revealed that miR156, miR159, miR160, miR164, and miR171 families are involved in response to *Pseudoperonospora cubensis* infection in cucumber (Jin and Wu, 2015), while miR156 and miR408 are involved in the response to viral infection in tabacum (Chen et al., 2017). In addition, some miRNA families such as miR159, miR164, miR169, and miR171 play important roles in regulation of the basal defense response during leaf rust and powdery mildew infection in wheat (Kumar et al., 2017). Thus, we proposed that these miRNA families might play a role in the response to *C. gloeosporioides* infection in tea plant.

Among the identified 239 known and 369 novel DEMs under *C. gloeosporioides* stress in this study, the majority were down-regulated, while others were induced under *C. gloeosporioides* stress. Many conserved miRNAs in our study showed similar expression patterns in response to disease infection across different plant species, suggesting functional conservation in this particular biological process. For instance, the expression levels of miR156, miR159, miR164, miR168, miR172, miR393, miR396, miR398, miR403, and miR472 were significantly altered in tea plant after infection; expression levels of these miRNAs also changed in response to fungal infection in cotton, rice, wheat, and pine (Xin et al., 2010; Yin et al., 2012; Li et al., 2014; Lu et al., 2007). Notably, our study initially identified some conserved miRNAs, which were responsive to *C. gloeosporioides* in tea plant, such as csn-miR3630-3p, csn-miR5368-p5, csn-miR7733, csn-miR7771-p5, and csn-miR9777. These miRNAs may contribute to biotic stress response in tea plant and are of interest for further investigation.

### *C. gloeosporioides*–responsive miRNAs Targeting Defense Responsive Genes

Plants conflict with the majority of the invaders like fungi and virus by activating intricate immune responses, which usually result in a disease resistance response. On the other hand, pathogens have normally developed habits to bypass plant defenses, and susceptibility to pathogens reappears. In addition to this random immune failure of the host, other immune-response independent processes allow further entry of the invading pathogen and contribute to plant pathogen susceptibility (Dobon et al., 2015). In this study, we found over 1,500 miRNA targets with diverse functions in response to *C. gloeosporioides*. For a better understanding, we propose a regulatory network in tea plant, derived from integrating our results with previous findings (**Figure 11**). Being a hemibiotrophic fungi, *C. gloeosporioides* initially act as biotrophic phase during which the host’s immune system and cell death are actively suppressed and allow invasive hyphae to spread throughout the infected tea plant tissues. This is followed by a necrotrophic phase during which toxins are secreted by the pathogen to induce host cell death and create tea plant susceptibility to this fungi. Time-course experiments carried out by Vargas et al. (2012) also revealed the biotrophic growth in susceptible maize leaves; the hemibiotrophic fungus *Colletotrichum graminicola* induced classical plant defense responses, such as the accumulation of ROS and phenolic compounds. They hypothesized that it is the switch to necrotrophy that enables the fungus to evade the plant immune system and allows pathogenicity.

Characteristic fungus–plant interfaces that accumulate effectors in biotrophy were previously reported in hemibiotrophs, the rice blast fungus *Magnaporthe oryzae* (Mosquera et al., 2009; Khang et al., 2010), and the crucifer anthracnose fungus *Colletotrichum higginsianum* (Kleemann et al., 2012). During the biotrophic phase, fungus must obtain nutrients from living host cells and tissues and secrete limited amounts of effectors to suppress the host immune system. PTI is the first layer of innate immunity and is initiated by plants in response to pathogen infection (Stergiopoulos and de Wit, 2009). C. *gloeosporioides* species exhibits a broad geographic range and has the capacity to cause severe damage on plants, resulting in large economic losses in agriculture (Rabha et al., 2016). Generally, *Colletotrichum* sp. delivered effector to the host was tightly coupled to early biotrophic expression of effector genes, which implies biotrophy-associated functions, such as the maintenance of biotrophy and/or suppression of postinvasion defenses until the switch to necrotrophy (Irieda et al., 2016). Likewise, during initial infection, *C. gloeosporioides* synthesize and secrete effector proteins, which actively pass signals to host. Activation of PRRs, such as leucine rich repeat (LRR) protein kinase, serine/threonine (Ser/Thr) protein kinase, and LRR receptor like Ser/Thr protein kinase by PAMPs, leads to phosphorylation and activation of LRR; this in turn leads to activation of a number of downstream signaling components for first line of PTI defense signaling (Jones and Dangl, 2006). Interestingly, our results showed that the LRR protein kinase can be targeted by csn-miR396b-p3/p5_2ss18AT19CT (down-regulated) and csn-miR5368-p5_1ss(3/4)TC (up-regulated). The Ser/Thr protein kinase can be targeted by three down-regulated miRNAs (csn-miR5368-p5_2ss15TC18CT, PC-3p-106557_16, and PC-3p-70583_26), and LRR receptor like Ser/Thr protein kinase can be targeted by csn-miR156a-p3_1ss10TA (up-regulated) and PC-3p-1581_1049 (down-regulated) after *C. gloeosporioides* infection in tea plant (**Supplementary Tables S5** and **S6**). Therefore, we provide evidence that these miRNAs function to target LRR protein kinase, Ser/Thr-kinase, and LRR receptor like Ser/Thr protein kinase receptors, thereby triggering a first line of PTI defense. The immune system enables the perception of the pathogen attack as the infection process starts, being the expression of susceptibility conditioned by the magnitude and/or timing of defense responses.

Plant susceptibility to hemibiotrophs is a genetically complex process and comprises the coordinated action of a wide range of hormones, including ET, JA, SA, and abcisic acid (ABA) (Thomma et al., 1998; Berrocal-Lobo et al., 2002; Adie et al., 2007; García-Andrade et al., 2011). Study by Diniz et al. (2017) has shown that there is a stronger activation of ERF1 gene at the beginning of the necrotrophic phase of hemibiotrophic fungi *Colletotrichum kahawae* in the susceptible variety of *Coffea arabica*, which suggests the involvement of ET in tissue senescence. ET pathway activation in the susceptible variety may be related with tissue damage promoted by the fungal necrotrophic phase. In this study, we found that a novel miRNA (PC-5p-66414_28) significantly targets ET insensitive 3, which activates the ET pathway; thereby we suggest that in later infection stages, *C. gloeosporioides* might deliver effectors or phytotoxins that manipulate the tea plant to produce ET for entering the necrotrophic stage of infection and therefore overcome plant defenses.

As a consequence of the biotrophic phase, fungi that require living host tissues fail to survive and infect plant. So, later, these fungi enter the necrotrophic phase, which mainly depends on ETI. ETI is triggered by the recognition of pathogen effectors by plant R proteins that leads to a hypersensitive response (HR) and localized host cell death (Jones et al., 2006). The largest class of R proteins belongs to the conserved family of nucleotide binding site LRR (NBS-LRR) proteins (Tameling and Takken, 2007). Rice blast fungus (*M. oryzae*) effectors are recognized by two divergently transcribed NBS-LRR genes, RGA4 and RGA5, which impart resistance against *M. oryzae* infection in rice (Cesari et al., 2013). The expression of tomato miR482, which targets some NBS-LRR protein genes, suppresses bacterial and viral pathogens (Shivaprasad et al., 2012). Notably, we identified NBS-LRR in tea plant as a potential target of csn-miR482b-p5_2ss9TA19AC and csn-miR3633a-3p_R-3_1ss7TA (**Supplementary Table 6**). We also found that more miRNAs are targeting the HR related PTI6 protein. HR at the site of infection also activates systemic acquired resistance (SAR), which provides protection against pathogens throughout the plant (Durrant and Dong, 2004; Chen et al., 2017). SAR requires the signal molecule SA, which induces the accumulation of PR proteins (Van Loon and Van Strien, 1999). Nonexpresser of PR gene1 (NPR1) is the central regulator of the SA signaling pathway and functions as a co-activator for SA-responsive genes. NPR1 monomers are then translocated into the nucleus where they interact with TGA-bZIP transcription factors (Zhou et al., 2000), leading to an activation of SA-dependent gene expression (Fan and Dong, 2002). In our study, we found that the transcriptional activator of PR genes, PTI6, was targeted by an up-regulated conserved miRNA (csn-miR159a-5p_L-1R-1) (**Supplementary Table 6**). Based on this result, we suspect that the PR gene transcription activator targeted by this miRNA should regulate PR genes, which encode antimicrobial proteins; thereby it is suggested that *C. gloeosporioides* enter the necrotrophic phase and activate R protein-mediated ETI to cause HR cell death, which leads to effector-triggered susceptibility (ETS). Our results emphasize the hypothesis that a cell wall integrity surveillance system evolved to sense the presence of a pathogen and to transduce signals into a rapid transcriptional reprogramming of the affected cell. This transcriptional reprogramming might serve to promote fungal growth to facilitate susceptibility of tea plant.

### *C. gloeosporioides*–Responsive miRNAs Targeting Transcription Factors

MiRNAs have important functions in plant stress responses by targeting multiple transcription factors, which, in turn, regulate the expression of various downstream genes involved in the stress response (Shukla et al., 2008). In our study, we found that some miRNAs and their corresponding target genes are involved in the regulation of transcription factors, hormone signaling, and crosstalk between defense-related signaling hormones (ABA, SA, JA, ET, and auxin) as well as phenylpropanoid biosynthesis; this is consistent with previous studies, which showed that SA signaling is an integral part of both the PTI and ETI defense responses (Yi et al., 2014, Chen et al., 2017). We also found that miRNAs target multiple transcription factors such as MYB, WRKY, NAC, and NF-YA. MYB is a transcription factor family in plants, which is involved in the regulation of a wide range of molecular events (Ambawat et al., 2013). Many studies suggest that PAMP triggers MYB induction, which would result in SA accumulation (Lee et al., 1995; Seo and Park, 2010). Our results showed that csn-miR828a, csn-miR858b_R-3, csn-miR858a_L-1R+1, and csn-miR6300-p3_1ss10AG target the MYB transcription factor, which is consistent with previous findings for heat response in cotton (Guan et al., 2014; Wang et al., 2016). WRKY is another well-known transcription factor family in plants, which is involved in various stress response networks (Banerjee and Roychoudhury, 2015). We found that WRKYs were targeted by down-regulation of the novel miRNA PC-5p-80764_22. NAC and its family members act as major transcriptional regulators of plant development and plant responses to biotic and abiotic stresses (Feng et al., 2014). Recently, the miR164 family was shown to target six NAC family members under various conditions of stress in several plant species (Bhardwaj et al., 2014; Feng et al., 2014). In addition, Jeyaraj et al. (2017) found that miR164 mainly targets NAC genes in response to biotic stress (geometrid attack) in tea plant; in accordance, we found that down-regulation of csn-miR164a and csn-miR164a_L+1_(1/2), which target NAC genes, was involved in the regulation of NACs. Members of the nuclear factor Y (NF-YA) transcription factor family have been reported to be key regulators of plant development, phytohormone signaling, and drought tolerance (Ren et al., 2016). Recently, Luan et al. (2015) found that the miR169 family members regulate the expression of NF-YA genes *via* transcript cleavage in response to abiotic stress in maize leaves. In addition, Ni et al. (2013) reported that NF-YA, a target gene of miR169, is a positive regulator of plant tolerance to drought stress. Interestingly, we observed significant up-regulation of csn-miR169i-p3_1ss6GA, csn-miR169e_1ss21GC, csn-miR169(a/q)_R-2, and csn-miR169x_R+1_2ss11TC21AC, as well as significant down-regulation of csn-miR169g-p3_1ss10CT, csn-miR169b, and csn-miR169e_R-3 under *C. gloeosporioides* stress in tea plant.

Some highly conserved pathogen-responsive miRNAs, including miR393, miR160, and miR167, play important roles in regulating perception and signaling of auxin (Yang et al., 2013). miR393 down-regulates transport inhibitor response 1 (TIR1) and represses transcription of auxin binding F-box proteins (Navarro et al., 2006), while miR160 and miR167 down-regulate five different ARF transcripts by guiding cleavage of their cognate mRNAs (Yang et al., 2013). It was previously demonstrated that many types of stresses, including bacterial and fungal infection, could up-regulate miR393 and repress auxin signaling by keeping TIR1 at a low level, thereby increasing AUX/IAA-ARF heterodimerization (Sunkar et al., 2012). However, we observed that csn-miR393a targeting TIR1 was down-regulated while ARF was targeted by an up-regulated miRNA [csn-miR7733-(p3/p5)_2ss12CT17CA] and five down-regulated miRNAs (csn-miR167k_R-3_1ss9CT, csn-miR160a, csn-miR160a_L+1, csn-miR160b, and csn-miR160c) after *C. gloeosporioides* infection. Thus, we speculated that miR393, miR160, and miR167 may work together, but may also involve other regulators to regulate the auxin pathway in tea plant during *C. gloeosporioides* infection.

### *C. gloeosporioides*–Responsive miRNAs Involved in MAPK Activation

The MAPK pathway is one of the most conserved signaling cascade pathways in plants and regulates a plethora of cellular processes including normal growth and development and plant defense responses against abiotic and biotic stresses (Nakagami et al., 2005). The PAMP receptor LRR protein kinase and ROS production cause downstream activation by phosphorylating a complete MAPK cascade (Suarez-Rodriguez et al., 2007). Thereafter, the activated MAPK regulates stress responses by phosphorylation of specific MYB and WRKY transcription factors (Andreasson et al., 2005). SA and various pathogen-derived elicitors were shown to induce the MAPKs and SA-induced protein kinase (SIPK) (Zhang and Klessig, 1997). Expression of constitutively active forms of MAPK and SIPK leads to multiple gene expression and HR-like cell death (Yang et al., 2001). Studies have reported that the silencing of MAPK attenuates plant resistance to bacteria and virus pathogen (Jin et al., 2002; Liu et al., 2004). Interestingly, it was observed that miR531 targets most members of the MAPK cascade gene family in *Oryza sativa* (Raghuram et al., 2014). We revealed in our study that csn-miR6173-p3_1ss4CT and PC-5p-167506_9 were up-regulated and targeted the MAPKs (**Supplementary Table S6****and****S7**). This finding suggests that these miRNAs are involved in the MAPK induced signaling cascade, thereby triggering the miRNA-mediated transcriptional regulation of WRKYs and MYB against *C. gloeosporioides* stress in tea plant.

### *C. gloeosporioides*–Responsive miRNAs Regulates Other Targets

Apart from various transcription factor genes, signal transducing genes, and kinases, *C. gloeosporioides* also target the ROS scavenging system in tea plant. Rapid production of ROS is the early change associated with pathogen infection in plants, which causes the development of the antioxidant system through a group of enzymes as well as non-enzymatic components, such as ascorbate and glutathione (Silveira et al., 2015). Increased activities of antioxidant enzymes under pathogen attacks are observed in several crops, such as sorghum (*Sorghum bicolor*), soybean (*Glycine max*), cucumber (*Cucumis sativus*), and common bean (*Phaseolus vulgaris*) (Datnoff et al., 2007; Resende et al., 2012; Polanco et al., 2014). We observed that among the reported genes encoding antioxidant enzymes, catalases isozyme1 (*CAT*) was targeted by three down-regulated miRNAs (csn-miR8757b-p5_1ss1TG, csn-miR396b-p3_2ss18AT19CT, and csn-miR396b-p5_2ss18AT19CT). *L-APX*, which encodes L-ascorbate peroxidase, was targeted by two up-regulated miRNAs (csn-miR408_L-1R+1 and csn-miR408-p3_2ss18GT19GT). *GR*, encoding glutathione reductase, was targeted by an up-regulated miRNA (PC-3p-58653_32) (**Supplementary Tables 6** and **7**). *GST*, encoding glutathione S-transferase, is known to play a key role in ROS detoxification and reduction in response to oxidative burst after pathogen infection (Bhattacharjee, 2012). Consistent with earlier results, the present study observed that csn-miR166g-5p_2ss5GA8GT, the miRNA that targets *GST*, was down-regulated. *COX*, which encodes cytochrome c oxidase, is a crucial component of the mitochondrial respiratory chain and is of utmost importance for providing cellular energy (Radin et al., 2015). A previous study reported that miR398 targets *COX* subunit V and helps plants to cope with various stresses (Sunkar and Zhu, 2004). Here, we found that *COX* subunit V was targeted by csn-miR398a (up-regulated); peroxidase was targeted by three conserved miRNAs (csn-miR166e-p5_2ss10GT17CT, csn-miR8005a-p5_1ss11AG, and csn-miR821b-p5_2ss14TG20AT) and two novel miRNAs (PC-3p-180347_8 and PC-3p-184859_7) (**Supplementary Tables 6** and **7**). Therefore, we inferred that miRNAs regulate antioxidant genes that are involved in the ROS responses in tea plant against *C. gloeosporioides* infection.

Apart from these, nitric oxide activates the SA biosynthesis pathway by inducing phenylalanine ammonia lyase (PAL), which is a key enzyme in biosynthesis of SA. PAL is one of the most extensively studied enzymes in the response pathways of plant biotic and abiotic stress (MacDonald and D’Cunha, 2007). Saravanakumar et al. (2007) have shown that the accumulation of peroxidase and PAL increases in response to pathogen infection in the tea plant. Studies have shown that *C. gloeosporioides* and *Colletotrichum camelliae* infection in various plants up-regulated the expression of PAL (Wang et al., 2016). In this study, we found that the expression of PAL was highly regulated by the miR477 family (csn-miR477g-p5, csn-miR477g-p5_1ss1CT, and csn-miR477g-p5_2ss1CT22CT) after *C. gloeosporioides* infection in tea plant (**Supplementary Table 6**). Thus, ROS scavenging system also plays an important role in *C. gloeosporioides* stress responses mediated by regulating the glycosylating hormones and secondary metabolites in tea plant. We speculate that during plant infection, the production and recognition of specific ligand peptides might act as pathogen effectors to facilitate the suppression of the plant’s immune system and orchestrate the reprogramming of the infected tissue so that it becomes a source of nutrients that are required by the pathogen to support its growth and development, which could contribute to the enhanced susceptibility. Thus, fungi might have adapted to these plant processes to improve the invasion of the host.

## Conclusions

In the present study, we used high-throughput sequencing to identify 485 conserved miRNAs and 761 novel miRNAs from mock- and *C. gloeosporioides*–inoculated tea plant leaves. Our results show that several important miRNAs are differentially expressed in *C. gloeosporioides*–infected leaves. Through degradome sequencing analysis, a total of 1,134 targets for conserved and 596 targets for novel miRNAs were identified. GO annotation revealed that the top ranked miRNA targeting genes were involved in diverse biological processes, including regulation of transcription, response to stress, biosynthetic processes, and signaling pathway. KEGG pathway analysis showed that the potential target genes of the miRNAs were mainly involved in translation, carbohydrate metabolism, signal transduction, energy metabolism, and amino acid metabolism. Moreover, negative correlations between expression levels of identified miRNAs and their corresponding targets were validated through qRT-PCR, and we verified the potential target genes (ARF, NFY, MYB75, MYB114, and STK1) for selected miRNAs by 5’RLM-RACE. These target genes were actively involved in the regulation of auxin pathway, ROS scavenging pathway, SA mediated pathway, receptor kinases, and transcription factors, which, in turn, actively intricate to various biological responses against *C. gloeosporioides* stress. A hypothetical model of the *C. gloeosporioides*–responsive miRNA regulatory network and their target genes in tea plant was derived from the data and illustrated schematically. These results will facilitate a comprehensive understanding of *C. gloeosporioides* stress in tea plant, and help to elucidate miRNA-mediated molecular mechanisms underlying tea plant responses to *C. gloeosporioides* and provide insights into the functional role of miRNAs and their targets in tea plant susceptible to this fungus.

## Data Availability

The sRNA read data sets supporting the results in this study have been deposited in the Gene Expression Omnibus (GEO) database; the accession number is GSE119728 (https://www.ncbi.nlm.nih.gov/geo/query/acc.cgi?acc=GSE119728). Tea transcriptome sequence data are deposited in NCBI Sequence Read Archive (SRA) under GenBank accession no. SRR1979118.

## Author Contributions

AJ participated in sample collection, performed the experiments and data analysis, interpreted the results, and drafted the manuscript. XW and SL participated in manuscript revision critically. SW, RZ, and AW participated in sample collection and qRT-PCR experiments. CW provided guidance on the experimental design and received funding. All authors read and approved the final manuscript.

## Funding

This work was partially supported by the National Key Research and Development Program of China (2018YFD1000601), the National Natural Science Foundation of China (31800585), the Special Innovative Province Construction in Anhui Province in 2015 (15czs08032), and the Special Project for Central Guiding Science and Technology Innovation of Region in Anhui Province (2016080503B024).

## Conflict of Interest Statement

The authors declare that the research was conducted in the absence of any commercial or financial relationships that could be construed as a potential conflict of interest.
